# Toxigenic effects of two benthic diatoms upon grazing activity of the sea urchin: morphological, metabolomic and *de novo* transcriptomic analysis

**DOI:** 10.1038/s41598-018-24023-9

**Published:** 2018-04-04

**Authors:** Nadia Ruocco, Susan Costantini, Valerio Zupo, Chiara Lauritano, Davide Caramiello, Adrianna Ianora, Alfredo Budillon, Giovanna Romano, Genoveffa Nuzzo, Giuliana D’Ippolito, Angelo Fontana, Maria Costantini

**Affiliations:** 1 Department of Biology and Evolution of Marine Organisms, Stazione Zoologica Anton Dohrn, Villa Comunale, 80121 Napoli, Italy; 2 Department of Biology, University of Naples Federico II, Complesso Universitario di Monte Sant’Angelo, Via Cinthia, 80126 Napoli, Italy; 3 Bio-Organic Chemistry Unit, Institute of Biomolecular Chemistry-CNR, Via Campi Flegrei 34, Pozzuoli, Naples, 80078 Italy; 4 Unità di Farmacologia Sperimentale, Istituto Nazionale Tumori “Fondazione G. Pascale”, IRCCS, Napoli, Italy; 5 Center of Villa Dohrn Ischia-Benthic Ecology, Department of Integrative Marine Ecology, Stazione Zoologica Anton Dohrn, P.ta S. Pietro, Ischia, Naples, Italy; 6 Department of Integrative Marine Ecology, Stazione Zoologica Anton Dohrn, Villa Comunale, 80121 Napoli, Italy; 7 Unit Marine Resources for Research, Stazione Zoologica Anton Dohrn, Naples, Italy

## Abstract

Diatoms are unicellular algae playing a key role as photosynthetic organisms in the world’s ocean food webs. The chemical ecology of planktonic diatoms is well documented, but few studies have reported on the effects of benthic diatoms on their consumers, also due to difficulties in the collection, quantification and massive culturing of benthic species. Here for the first time we investigate the effects of feeding on two abundantly occurring benthic diatoms, *Nanofrustulum shiloi* and *Cylindrotheca closterium*, isolated from the leaves of the seagrass *Posidonia oceanica*, on the sea urchin *Paracentrotus lividus*. Adult *P. lividus* were fed for one month on diets of either one of the two diatoms and on the green alga *Ulva rigida*, used as a feeding control. By combining morphological, metabolomic and *de novo* transcriptomic approaches, we demonstrate toxigenic effect on embryos generated by females fed with these benthic diatoms. Furthermore, chemical analysis reveal the presence of polyunsaturated aldehydes only for *N. shiloi*, and a high production of other oxylipins (cytotoxic compounds on their grazers and on cancer cell lines) for both diatoms, including some additional peaks not correlated to the canonic oxylipins commonly observed in planktonic diatoms. These findings open new perspectives in the study of diatom secondary metabolites influencing their grazers.

## Introduction

Diatoms are unicellular eukaryotes, representing one of the largest and ecologically groups and exclusively depositing biogenic silica. The siliceous wall is transparent, allowing the entrance of the light, and perforated, making possible the diffusion and excretion of materials^1^. They contribute about 20% of global photosynthetic fixation of carbon (about 20 Pg carbon fixed per year), which is more than all the world’s tropical rainforests, also playing important roles on earth and in oceans as oxygen synthesizers and biomass sources^2^. Functionally, diatoms are single cells but they can appear as filaments, chains, or colonies, and they are abundant in nearly all aquatic habitats, living either in the water column (planktonic species) or attached to any single substratum (benthic species). Moreover, diatom morphology can be considered as an additional environment assessment tool to the biological indices to evaluate for example the anthropogenic eutrophication effects^3^.

Diatoms have been regarded as beneficial to the growth and survival of primary consumers such as planktonic and benthic filter feeders. However, many planktonic diatoms have been discovered to produce a wide range of oxygenated fatty acid derivatives (called oxylipins)^4,5^ that affect diatom growth^6,7^ or have negative effects on the reproduction and development of several marine invertebrates, such as copepods^8,9^, sea urchins^10–15^ and sea stars^16^, polychaete worms^17^ and ascidians^16,18^. Moreover some oxylipins, the diatom derived polyunsaturated aldehydes, activated cell death in human cancer cell lines^19^.

In contrast to the chemical ecology of planktonic diatoms that is well documented^20,21^, few studies have investigated the chemistry of benthic diatoms also due to difficulties in their isolation, quantification and cultivation in plates, with respect to planktonic species^22^. Diatoms are characterized by rapid and extensive colonization of benthic substrate occurs through deposition of stalks, creating thick mats and exhibiting a strong dependence on hydrodynamic conditions and requiring stable substrates to establish a population^23,24^.

Some *Cocconeis* species have been shown to induce sex reversal in the shrimp *Hippolyte inermis*, a benthic opportunistic herbivore, but the chemicals that induce sex reversal remain unknown^25–27^. Other benthic diatoms produce volatile organic compounds (VOCs) similar to those produced by planktonic diatoms that induce behavioural changes in several species of macrinvertebrates^28^, but studies on the metabolic effects of fast-growing benthic diatoms on benthic invertebrates are still largely lacking^29–31^.

Using morphological, metabolomic and *de novo* transcriptomic approaches here, for the first time, the effects of two abundantly occurring benthic diatoms, *Nanofrustulum shiloi* and *Cylindrotheca closterium*, isolated from the leaves of the seagrass *P. oceanica*, were investigated on the reproductive success of the sea urchin *Paracentrotus lividus*. Sea urchins were fed for one month on these two benthic diatoms in a microcosm, using the green alga *Ulva rigida* as a feeding control. The development of the embryos produced after feeding on these diets were then followed until the pluteus stage (48 hours post fertilization). In addition, gonadic tissues from these adults were analyzed by ^1^H-NMR metabolomics. Finally, molecular approaches were applied to investigate the toxic effects of the benthic diatoms, by generating a *de novo* transcriptome assembly and annotation of *P. lividus* to identify differentially expressed genes. Fifty genes, belonging to different functional classes, were also followed using Real-Time qPCR to detect if the expression level of these genes was modulated by feeding on the benthic diatoms.

## Results

### Morphological and molecular characterization of benthic diatoms

**Figure 1:**
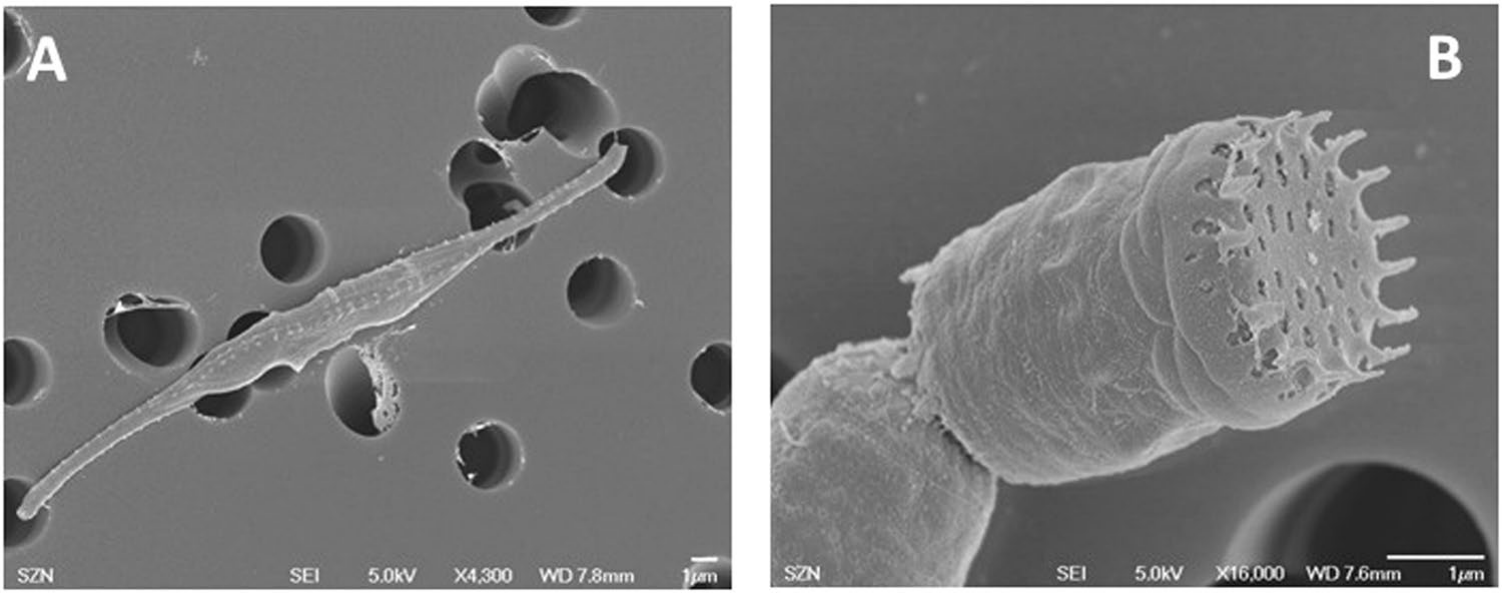
Scanning electron micrographs (SEM) of (**A**) *C. closterium* and (**B**) *N. shiloi* isolates. Scale bar = 1 μm.

SEM observation revealed that the first diatom isolated was about 50 μm in length, needle like and thin in shape, the two ends of the cell extended far from the centre of the cell; the cells showed spiral twist of the raphe system, which is characteristic for *Cylindrotheca closterium* (Fig. 1A). Molecular analysis of 18S rRNA gene amplified from the purified alga showed that it was closer (99%) to *C. closterium* than to other species. The second benthic diatom was characterized by rectangular frustules, forming chains linked by interlocking marginal spines characteristic of *Nanofrustulum shiloi* (Fig. 1B). This morphological result was also confirmed by 18S rRNA gene, showing 99% identity to *N. shiloi*.

### Feeding experiments

The biomass of benthic diatoms fed to sea urchin replicates was calculated to be 1.6 pg C cell^−1^ for *C. closterium* and 1.8 pg C cell^−1^ for *N. shiloi*^32^.

**Figure 2:**
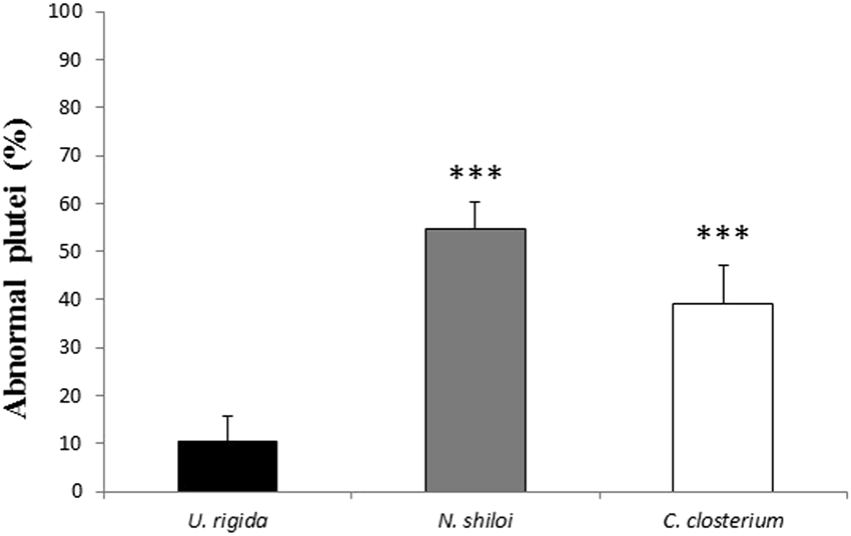
Percentage of abnormal plutei (%; at 48 hours post fertilization) of *P. lividus* sea urchin embryos spawned from adults fed for one month with *U. rigida*, *N. shiloi* and *C. closterium* (*** with a p-value < 0.001, Student’s t-test, GraphPad Software Inc., San Diego, CA, USA).

After one month of feeding, eggs and sperms were collected from sea urchins fed with *U. rigida*, *N. shiloi* and *C. closterium*. As soon as fertilization occurred, fertilization success and the time to reach first mitotic cleavage to obtain two blastomeres was measured in comparison with embryos deriving from sea urchins collected in the field at the beginning of the experiments (t0) (Table 1). At time 0 100% fertilization and first cleavage were obtained with all diets. Embryonic development was then followed until the *pluteus* stage; morphological observations showed that the percentage of abnormal embryos was higher in sea urchins fed on *C. closterium* and *N. shiloi* for one month (p < 0.0001) in comparison to the control diet (Fig. 2). In particular, both *C. closterium* and *N. shiloi* induced the same malformations, which principally affected the arms, spicules and apices, in comparison with control embryos (Supplementary Fig. S5). To confirm that sea urchins had really fed on diatoms, the content of fecal pellets was also analyzed by means of SEM. These observations showed the presence of *C. closterium* silica frustules in the fecal pellets (reported in the Supplementary Fig. S6 as an example), confirming that sea urchins have effectively eaten the diatoms.

**Table 1:** Percentage of fertilization, first cleavage (two blastomeres), normal plutei and malformed plutei in the embryos from sea urchins *P. lividus* collected in the field at the beginning (t0) and after one month of feeding with *U. rigida*, *C. closterium* and *N. shiloi*.

	t0	*U. rigida*	*C. closterium*	*N. shiloi*
Fertilization	100	100	100	100
First cleavage	100	100	100	100
Normal plutei	90	89.4	45.3	60.9
Malformed plutei	10	10.6	54.7	39.1

### ^1^H-NMR analysis of metabolites and lipids from sea urchin gonads

^1^H-NMR spectra were obtained from aqueous extracts of gonad tissues from five adult sea urchins fed with *U. rigida* (control), *N. shiloi* and *C. closterium*. The primary peaks in the spectra were assigned to individual metabolites (Supplementary Table S2).

Some metabolite classes were identified including acetoacetate, ATP, choline, lactate, glucose and amino acids (valine, leucine, isoleucine, alanine, threonine, arginine, lysine, glutamate, glutamine, aspartate, glycine, tyrosine, phenylalanine, tryptophan and histidine).

**Figure 3:**
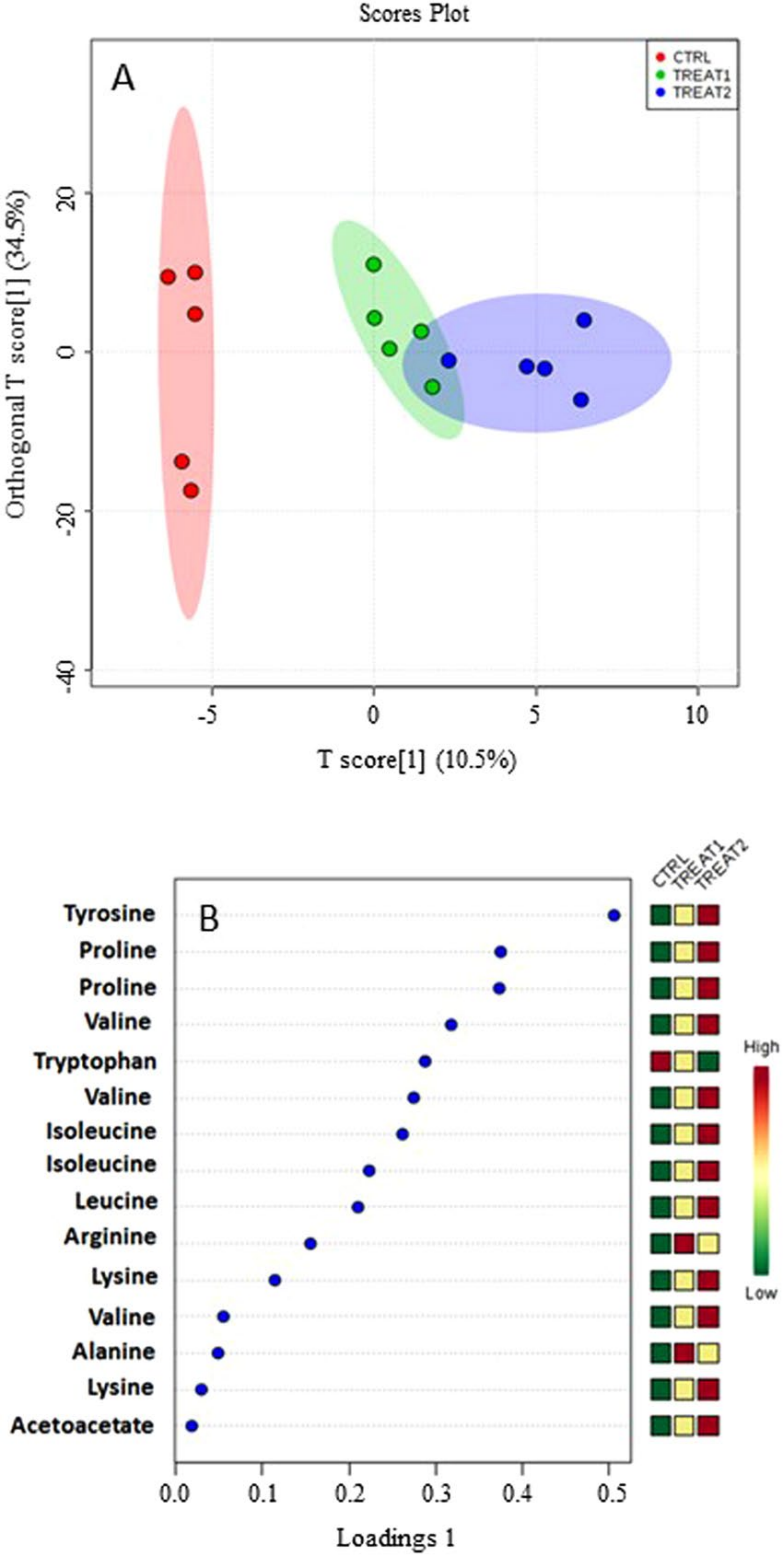
OPLS-DA (**A**) and Loading (**B**) plots (where the metabolites increased or decreased) of aqueous extracts from gonad tissues from adults sea urchin *P. lividus* after one month of feeding with *U. rigida* (used ad feeding control, reported as CTRL), *N. shiloi* (reported as TREAT1) and *C. closterium* (reported as TREAT2).

OPLS-DA plot (45% of the total variance) showed that the control and two treated groups clustered in separate classes, with a slight overlap between the two treated groups (Fig. 3A), suggesting the presence in these three groups of statistically different levels of metabolites (Fig. 3B). In particular: (i) the levels of acetoacetate and of six amino acids such as tyrosine, proline, valine, isoleucine, leucine and lysine, and acetoacetate were higher in the *N. shiloi* group when compared to the control group, and (ii) their levels were even higher in the *C. closterium* group when compared to the *N. shiloi* group. On the other hand, the levels of tryptophan decreased after feeding on both benthic diatoms and were lower in the *C. closterium* group compared to the *N. shiloi* group. Finally, the levels of arginine and alanine were higher in the treated groups when compared to the control group, and, higher in the *N. shiloi* group compared to the *C. closterium* group.

**Figure 4:**
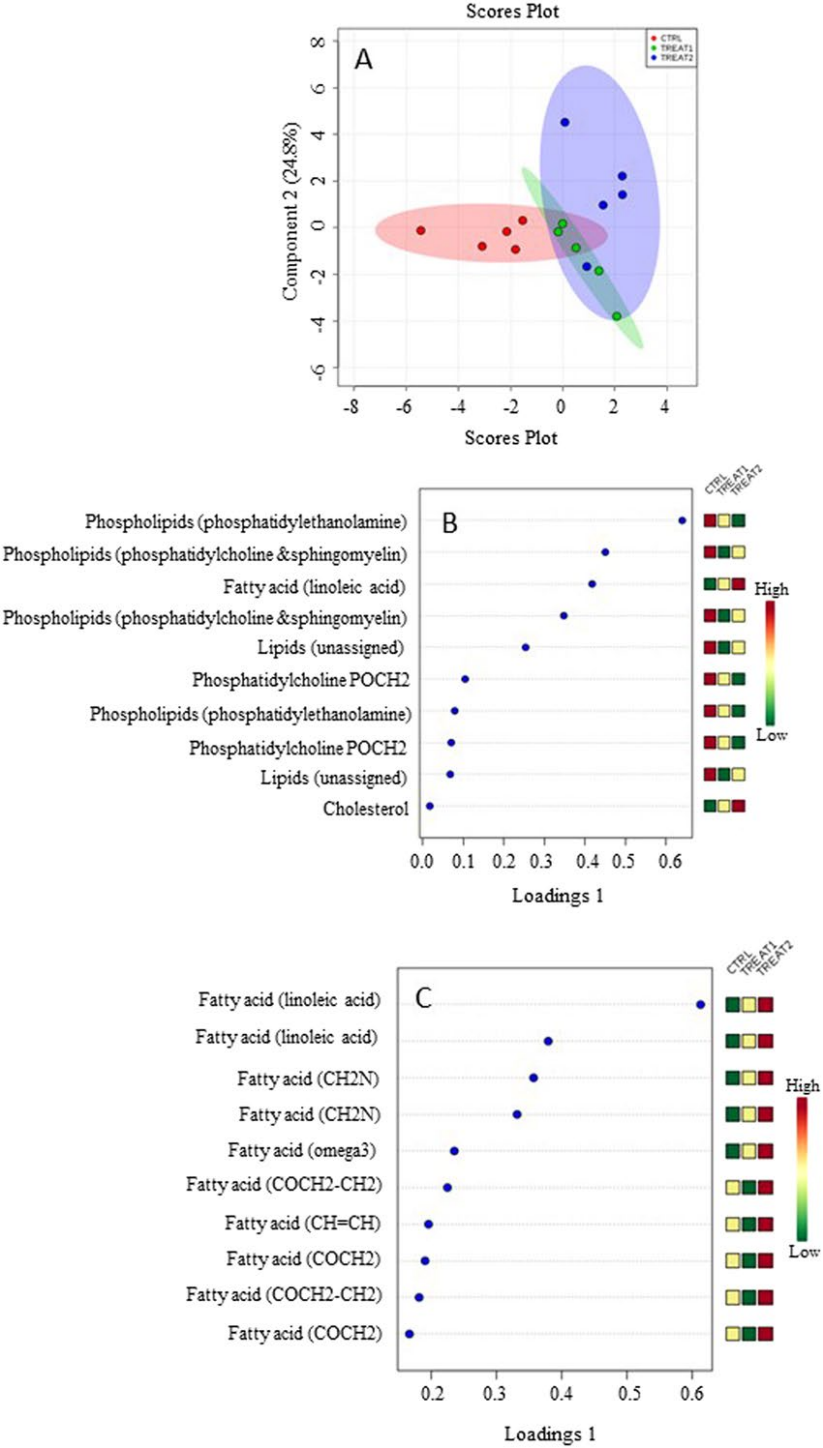
OPLS-DA (**A**) and Loading plots of (**B**) lipids and (**C**) fatty acids from lipophilic extracts of gonad tissues from adults sea urchin *P. lividus* after one month of feeding with *U. rigida* (used as feeding control, reported as CTRL), *N. shiloi* (reported as TREAT1) and *C. closterium* (reported as TREAT2).

^1^H-NMR spectra were also obtained from lipophilic extracts of gonad tissues. As shown in Supplementary Table S3, phosphatidylethanolamine, phosphatidylcholine, sphingomyelin, linoleic acid, cholesterol and other unassigned lipids were found in the gonads of *P. lividus*. OPLS-DA plot (38.1% of the total variance) showed that the control and two treated groups clustered in separate classes with a slight overlap between the control group and the two treated groups (Fig. 4A), and a total overlapping of the *N. shiloi* and *C. closterium* groups. These data suggest the presence in these three groups of statistically different levels of metabolites between the control and the two treated groups, and metabolites with similar levels between the two treatments. In fact: (i) the levels of linoleic acid and cholesterol were higher in the *N. shiloi* group when compared to the control group, and (ii) their levels were even higher in the *C. closterium* group when compared to the *N. shiloi* group. The levels of phosphatidylcholine, sphingomyelin and other lipids decreased after feeding on both benthic diatoms and were lower in the *N. shiloi* group compared to the *C. closterium* group; whereas the levels of phosphatidylethanolamine and phosphatidylcholine POCH_2_ were lower in the *N. shiloi* group compared to the *C. closterium* group (Fig. 4B). Considering only fatty acids, some fatty acids, such as linoleic acid and other fatty acids were higher in the *N. shiloi* group and even higher in the *C. closterium* group when compared to the *N. shiloi* group (Fig. 4C). On the other hand, other fatty acids decreased in *N. shiloi* and increased in *C. closterium* groups, with respect to the control group.

### Transcriptomic assembly

The raw assembled transcriptome included almost 121 Mbp in 192493 transcripts grouped in 126941 genes. The mean GC content was 41.37%. The average and median contig lengths were 357 bp and 627 bp, respectively. The N50 was 920 bp. All analyses were conducted on *de-novo* assembled genes and the biological replicates clustered according to the experimental design.

**Figure 5:**
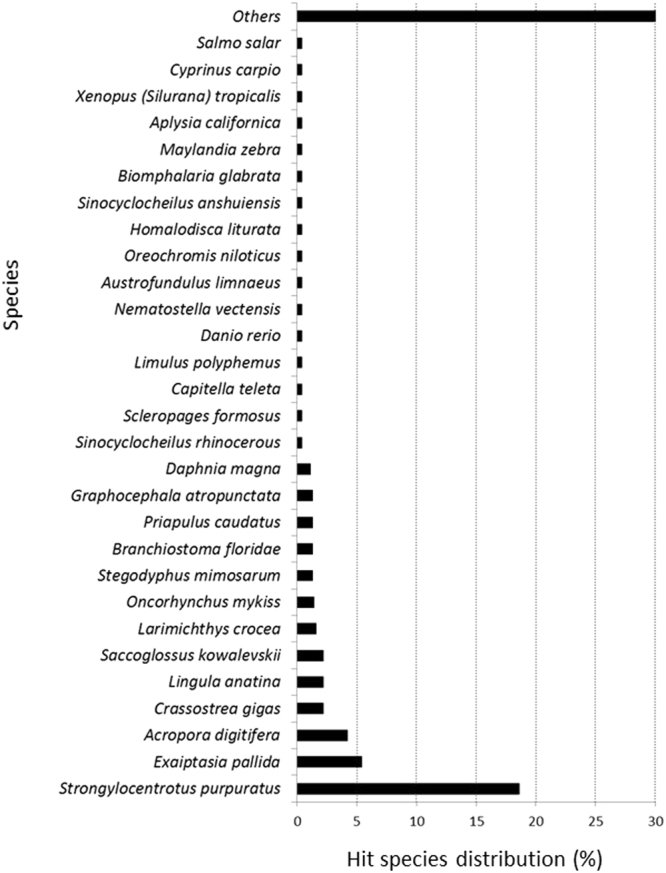
Blastx top hit species distribution of matches with known sequences.

Figure 5 reports the BLASTx top hit species distribution of matches with known sequences and indicates that the majority of *P. lividus* contigs (reads) showed the highest homology with *Strongylocentrotus purpuratus* (18.6%). The other most represented species included *Exaiptasia pallida* (5.4%), *Acropora digitifera* (4.2%), *Crassostrea gigas* (2.2%), *Lingula anatina* (2.2%) and *Saccoglossus kowalevskii* (2.2%). All alignments were carried out setting the E-value thresholds at a value of ≤1e-5.

### Differentially expressed genes in *P. lividus* plutei after feeding experiments (RNA-seq)

Differentially expressed genes were identified between the three conditions: embryos at the pluteus stage spawned by adults fed for one month with *N. shiloi* and *C. closterium*, compared to those fed with *U. rigida* as control, with each diet including three biological replicates. Using the R Bioconductor package DESeq. 2, the number of differentially expressed (DE) genes were count in pairwise comparisons, discriminating those that were up and down regulated. The total number of DE genes were: (i) 1285 between plutei from sea urchins fed with *N. shiloi* compared to controls fed with *U. rigida*; (ii) 2386 between plutei from sea urchins fed with *C. closterium* compared to control; (iii) 303 between plutei from sea urchins fed with *N. shiloi* compared with those fed with *C. closterium*. For all these genes the following DE annotated genes were found, considering FDR ≤0.05 fold change >1.5 for up regulated and fold change < −1.5 for down regulated: (i) in the case of plutei spawned from sea urchins fed with *N. shiloi*, 217 total genes were identified, of which 113 were upregulated (with a range of fold-changes between 1.8 and 3.6) and 104 downregulated (with a range of fold-changes between −1.7 and −12) compared to the control. Of these, some genes showed very high values of fold changes, such as the two downregulated genes *sperm flagellar 2* and *centrosomal of 170* *kDa B isoform X7*; (ii) in the case of plutei from sea urchins fed with *C. closterium*, 670 total DE genes were identified, of which 541 transcripts were upregulated (with a range of fold-changes between 1.7 and 20) and 129 downregulated (with a range of fold-changes between −1.5 and −10). Also in this case, some genes showed very high values of fold changes: the downregulated genes *vacuolar sorting-associated 13C- partial*, *centromere W* and *uncharacterized protein LOC589705* and the upregulated genes *cleavage stimulation factor subunit 1*, *fibrocystin-L* and *cAMP-responsive element-binding -like 2*. Furthermore, comparing the two treatments with *N. shiloi* and *C. closterium*, 303 DE genes were found, of which 177 upregulated genes (with a range of fold-changes between −1.7 and −30) and 129 downregulated (with a range of fold-changes between −1.6 and −20). Two genes were strongly downregulated, *CD9 antigen* and *leukocyte elastase inhibitor-like isoform X3* and two strongly upregulated, *isocitrate dehydrogenase [NADP] mitochondrial-like* (35.8-fold) and *peptidyl-prolyl cis-trans isomerase E*.

**Figure 6:**
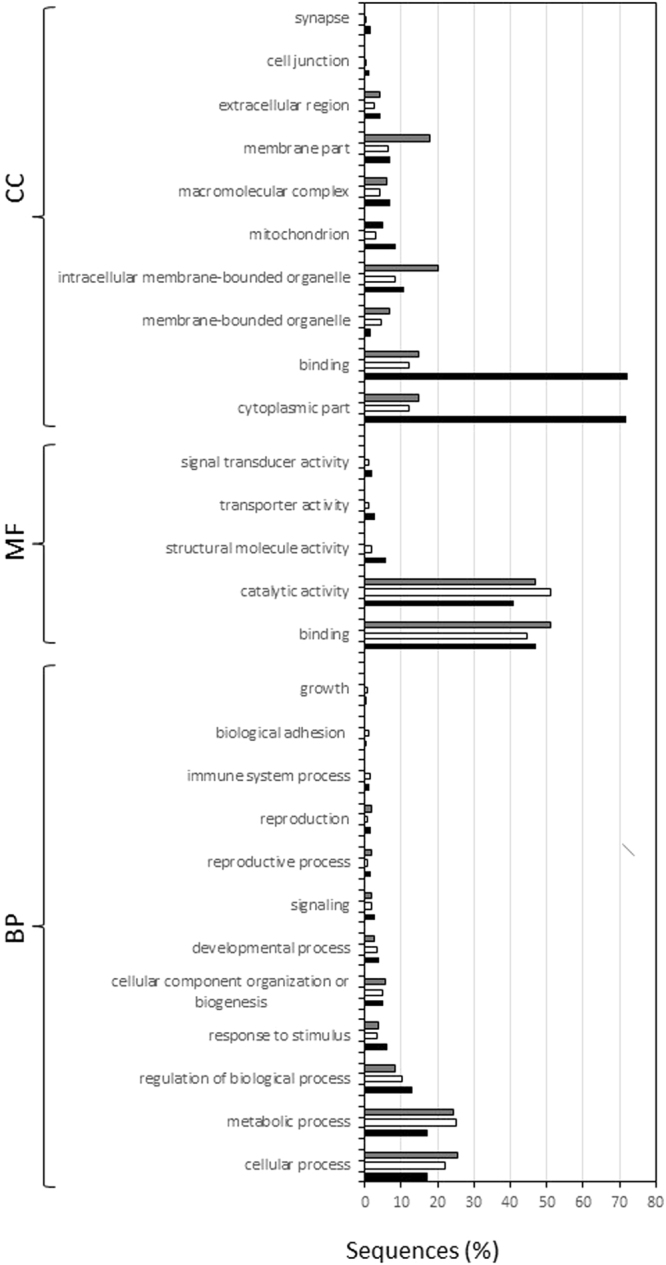
Overrepresented GO terms of sea urchin plutei after feeding experiments with the two benthic diatoms, *N. shiloi* and *C. closterium*, in comparison with *U. rigida* (feeding control), in the three major functional categories: biological process (BP), molecular function (MF) and cellular component (CC). The tree bars represent: black bar “control versus *N. shiloi*”, white bar “control versus *C. closterium*” and grey bar “*N. shiloi* versus *C. closterium*”.

To identify the pathways in which these genes were involved, a GO term enrichment analysis was performed using DE genes (Fig. 6). Twenty-seven GO terms were enriched including 12 in BP followed by 10 in CC and 5 in MF (p < 0.05). Overrepresented GO categories included binding, catalytic activity, cellular processes, metabolic processes and regulation of biological processes.

### Comparison of *de novo* transcriptome of *P. lividus* with *S. purpuratus*

Since the majority of *P. lividus* contigs (reads) showed the highest homology with the sea urchin *S. purpuratus* (see Fig. 5), *de novo* transcriptome of *P. lividus* was aligned with that of *S. purpuratus* (available at http://www.spbase.org/SpBase/search/)^33^. In the case of plutei from sea urchins fed with *N. shiloi*, 18 DE genes were identified, of which 11 were upregulated (with a range of fold-changes between 1.5 and 2.2) and 7 downregulated (with a range of fold-changes between −1.5 and −1.8) compared to the control. In the case of plutei from sea urchins fed with *C. closterium*, 41 DE genes were identified, of which 17 were upregulated (with a range of fold-changes between 1.5 and 2.2) and 24 downregulated (with a range of fold-changes between −1.5 and −2.3).

### Effects of *N. shiloi* and *C. closterium* on sea urchin embryos with Real time qPCR

**Figure 7:**
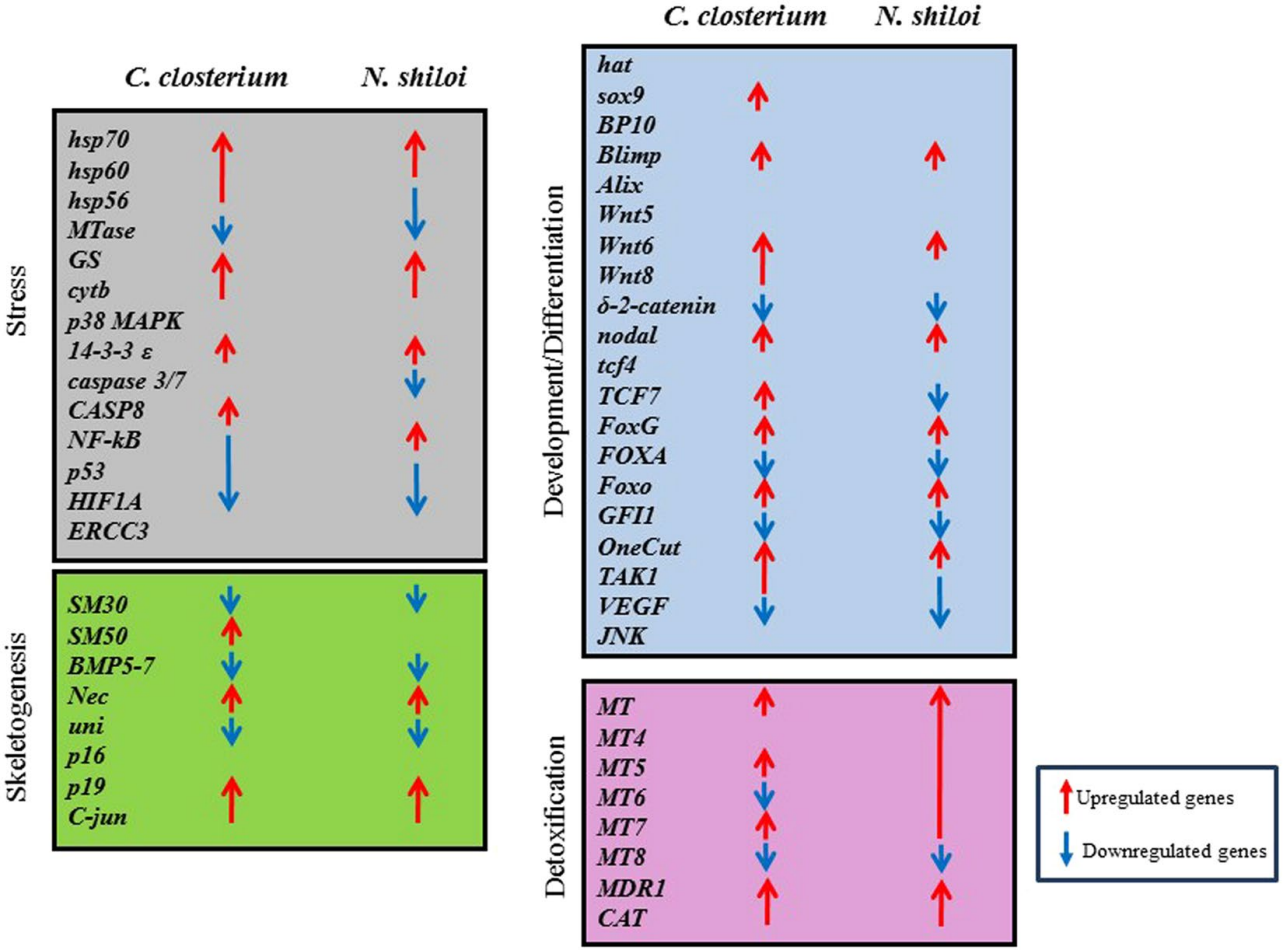
Synopsis of the patterns of up- and downregulation of different classes of genes in sea urchin embryos spawned from adults fed for one month with the two benthic diatoms *N. shiloi* and *C. closterium* (see also Supplementary Table S4 for the values and Supplementary Figs S7–S8).

Fifty genes, having key roles in different functional processes, were followed by Real Time qPCR (Supplementary Fig. S7)^11–13,34^. At the pluteus stage (48 hpf; Fig. 7, Supplementary Fig. S8 and Supplementary Table S4) *C. closterium* and *N. shiloi* had several common molecular targets.

- Stress: both benthic diatoms upregulated the expression levels of *hsp70*, *hsp60*, *GS*, *cytb*, *14–3–3ε* and downregulated *MTase*, *HIF1A* and *p53*. *C. closterium* and *N. shiloi* differentially affected the *hsp56* gene and *Nf-kB*. Moreover, *C. closterium* upregulated the genes *CASP8* whereas *N. shiloi* dowregulated *caspase 3/7* with respect to the control.
- Genes involved in skeletogenesis: both diatoms downregulated *SM30*, *BMP5–7* and *uni*; on the other hand, both species upregulated *Nec*, *p19* and *Jun*. Moreover, *C. closterium* increased the expression level of *SM50* gene.
- Development and differentiation: common molecular targets were *Blimp*, *Wnt6*, *nodal*, *FoxG*, *Foxo*, *OneCut/Hnf6*, *FOXA*, *VEGF*, δ-2-catenin and *GFI-1*. These two diatoms differentially regulated other genes, as in the case *TCF7* and *TAK1* genes, upregulated by *C. closterium* and downregulated by *N. shiloi*. *C. closterium* also affected the expression levels of two other genes of this functional class, upregulating *sox9* and *Wnt8*.
- Detoxification: *C. closterium* and *N. shiloi* increased the expression levels of *MT*, *MT5*, *MT7*, *MDR1* and *CAT* and decreased that of *MT8*. Furthermore, the two diatoms differently expressed the *MT6* gene; *N. shiloi* also increased the expression level of *MT4*.

## Discussion

Feeding experiments reported here revealed a noxious effect of two benthic diatom species (*C. closterium* and *N. shiloi*) on embryos spawned from adult sea urchins fed for one month on these diets. Embryos showed morphological malformations, affecting the arms, spicules and apex. These malformations were the same as those observed when sea urchin embryos were treated with some oxylipins, such as polyunsaturated aldehydes (PUAs, decadienal, heptadienal and octadienal) and hydroxyacids (5- and 15-HEPE), produced by planktonic diatoms^12,14^. These oxylipins have been shown to affect embryonic development in several marine invertebrates, including the sea urchin *P. lividus*^9,12–14,16^. Volatile organic compounds (VOCs), including PUAs, have been identified in several freshwater benthic diatoms, such as *Achnanthes biasolettiana*^35^, *Gomphonema parvulum*, *Amphora veneta*, *Fragilaria* sp. and *Melosira varians*^36^, but not much is known on the effects of these compounds on grazing invertebrates. Pezzolesi *et al*.^37^ highlighted the production of PUAs in three diatoms commonly occurring in the microphytobenthic communities in temperate regions, *Tabularia affinis*, *Proschkinia complanatoides* and *Navicula* sp. The existence of a large family of PUAs, including some with four unsaturations, such as decatetraenal, undecatetraenal and tridecatetraenal, has been observed.

Feeding on these diatoms also increased the levels of some lipids in the gonads of *P. lividus*. In particular, we observed a strong increase in linoleic acid and cholesterol, compared to sea urchins fed an *Ulva* diet. Gonads of this sea urchin are a rich source of PUFAs^38,39^ and linoleic (18:2n-6) and α-linolenic (18:3n-3) acids are found in higher proportions in the gonads of *P. lividus*^40^. The increased lipids in *P. lividus* gonads in our experiments could be due to the diet they were fed upon since diatoms are known to be rich in lipids, mainly PUFAs, usually comprising up to 15–25% of dry biomass^41–43^. *C. closterium* is particularly rich in 16:0 and 16:1 (n-7) fatty acids that can comprise up to 64% of the total lipids^44^. These findings also indicate an increase in the aqueous (polar) phase of the amino acids tyrosine, proline, valine, isoleucine, leucine and lysine in the gonads of sea urchins fed with *C. closterium* with respect to those fed with *N. shiloi*. Lower levels of these amino acids were found in control gonads from adults fed with *U. rigida*. Sea urchin gonads are rich in essential amino acids such as phenylalanine, threonine, valine, lysine leucine, isoleucine and histidine (about 32.1% of total amino acids) which cannot be synthesized *de novo* by the organism and must thus be supplied by the diet. In addition, the level of acetoacetate increased in the gonads of adults fed with *C. closterium* with respect to *N. shiloi*. Diatoms, like other photoautotrophs synthesize a wide range of amino acids for building proteins and other compounds^45^. The increase in essential amino acids (valine, isoleucine, leucine and lysine) after feeding with both benthic diatoms could be ascribed to the two diatom diets. Diatoms are known to produce high levels of leucine and fairly abundant quantities of lysine^45,46^. Specific data on the amino acidic and proteic compositions of the two benthic diatoms used in this study are not available, but it is known that benthic diatoms belonging to the genus *Chylindrotheca* are rich in proteins^47^. To date, knowledge on proteins and amino acid composition of gonads, eggs and larvae of echinoids are scarce and the possibility to modify their profiles through diet manipulations is relatively unknown. For example, Gago *et al*.^48^ found very few differences in the protein content of *P. lividus* eggs, prisms and pre-plutei and the amino acid composition of eggs from captive broodstock fed prepared diets or those obtained from wild broodstock. However, these results indicate that diet can significantly affect lipid, amino acid and protein levels of cultured sea urchins.

**Figure 8:**
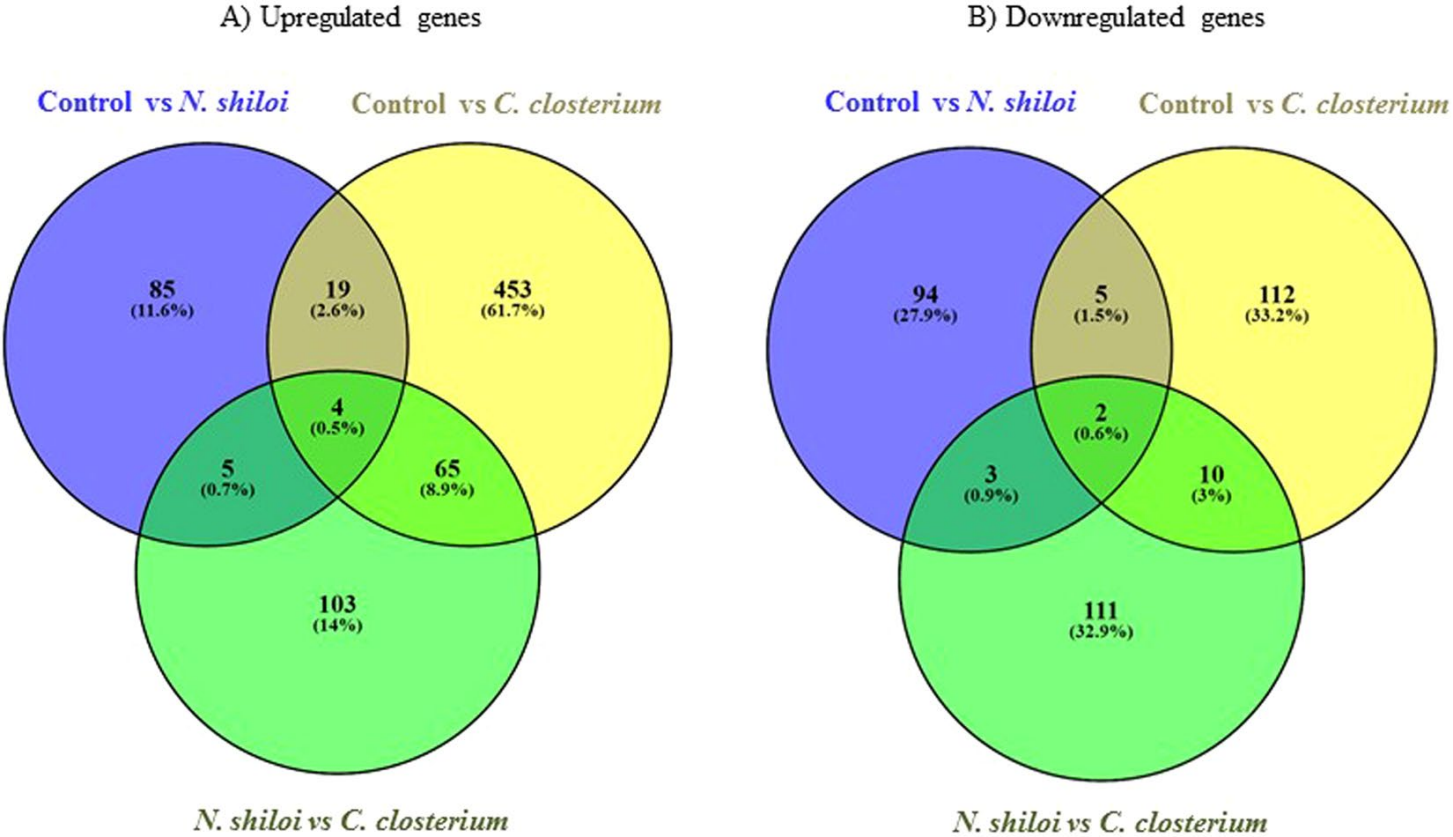
Venn diagrams considering upregulated genes and downregulated genes comparing the groups “control (*U. rigida*, feeding control) versus *N. shiloi*”, “control versus *C. closterium*” and “*N. shiloi* versus *C. closterium*”. *N. shiloi* and *C. closterium* induced an increase in the expression of 85 (11.6%) and 453 (61.7%) genes, respectively, compared to the control diet (*U. rigida*); they also induced the downregulation of 94 (27.9%) and 112 (33.2%) genes, respectively. The two diatoms had a few common targets (see also Supplementary Table S5 for the names of the common genes). In fact, in the case of up-regulated genes we found 19 common genes (2.6%) comparing the groups “control versus *N. shiloi*” and “control versus *C. closterium*”; 4 common genes (0.5%) comparing the groups “control versus *N. shiloi*”, “control versus *C. closterium*” and “*N. shiloi* versus *C. closterium*”; 5 common genes (0.7%) “control versus *N. shiloi*” and “*N. shiloi* versus *C. closterium*”; 65 common genes (8.9%) comparing “control versus *C. closterium*” and “*N. shiloi* versus *C. closterium*”. Considering the down-regulated genes, we found 5 common genes (1.5%) comparing the groups “control versus *N. shiloi*” and “control versus *C. closterium*”; 2 common genes (0.6%) comparing the groups “control versus *N. shiloi*”, “control versus *C. closterium*” and “*N. shiloi* versus *C. closterium*”; 3 common genes (0.9%) “control versus *N. shiloi*” and “*N. shiloi* versus *C. closterium*”; 10 common genes (3.0%) comparing “control versus *C. closterium*” and “*N. shiloi* versus *C. closterium*”.

Another interesting result is the large-scale genomic information on *P. lividus* generated in this study. Analysis of differentially expressed (DE) genes indicated that metabolic, cellular, reproductive, developmental, immune system, biological regulation and response to stimuli and biological adhesion processes were all affected by benthic diatoms. The two diatoms targeted different genes and had a few common targets (Fig. 8 and Supplementary Table S5).

The interpretation of *de novo* transcriptomic results were also improved with the analysis by Real Time qPCR of a set of fifty genes, previously used to study the response of *P. lividus* embryos to oxylipins produced by planktonic diatoms. These genes have key roles in different functional processes such as stress response, skeletogenesis, embryonic development, cell differentiation, morphogenesis and detoxification processes (see Supplementary Fig. S7). New results show that all these genes were common molecular targets for *N. shiloi* and *C. closterium*, with the only exception of *p38 MAPK*, *ERRC3*, *hat*, *BP10*, *p16* and *Wnt5*. Some of these targeted genes were common to the genes from RNA-seq, confirming transcriptomic results (see Supplementary Fig. S9 for some examples). For example, using Real Time qPCR we found that *N. shiloi* upregulated the expression level of *FoxG* gene (see Supplementary Table S4). This gene was also upregulated by *N. shiloi* in the comparison of *de novo* transcriptome of *P. lividus* with *S. purpuratus*. Furthermore, Real Time qPCR revealed that *cytb*, *hsps60* and *hsp70* were upregulated by both *N. shiloi* and *C. closterium*. *De novo* transcriptome analysis showed that *metallotionein*, *cytochrome c*, *cytochrome P450* and several *heat shock proteins* (*heat shock cognate 71*, *DnaJ*, *heat shock partial*, *hsp90*) were also upregulated with both diatoms. The genes *catenin alpha-2* was down-regulated and *14-3-3 epsilon* upregulated in the *de novo* transcriptomic comparison between the two diatoms, and the data validated using Real Time qPCR.

All together these molecular results, supported by morphological findings, revealed that the majority of malformations affected the skeleton, the developmental plan and differentiation of sea urchin embryos. In fact, several genes belonging to the skeletogenic, developmental and differentiation classes were affected by the ingestion of benthic diatoms. Even if we did not observe differences between the two diatom diets at the morphological level, the molecular results (and mainly the *de novo* transcriptome) suggest that the toxic effect of feeding of *P. lividus* on the diatom *C. closterium* was higher than that of *N. shiloi*.

This study is the first demonstration of the toxic effects of benthic epiphytic diatoms on embryos and larvae of the sea urchin *P. lividus* due to the feeding of adults during gonadal maturation. The effects may be considered comparable to those previously demonstrated in planktonic copepods fed on plankton diatoms^49^. Since there is few information available on chemical compounds from benthic diatoms, further studies are necessary to identify possible putative compounds responsible for the observed toxic effects on sea urchins. Preliminary investigations have been performed to test if these diatoms produce oxylipins, as in the case of planktonic species. GC-MS (Gas Chromatography-Mass Spectrometry) analysis revealed the presence of PUAs only for *N. shiloi* (data not shown). On the contrary, LC-MS (Liquid Chromatography Mass Spectrometry) profiles of methylated samples showed a high production of oxylipins, in particular hydroxy derivatives of eicosapentaenoic acid (EPA). In fact, MS spectra from both diatoms revealed the presence of two compounds: hydroxyeicosapentaenoic acids (HEPEs) and hydroxy-epoxy-eicosatetraenoic acids (HepETEs) (data not shown). In addition, both benthic diatoms showed some additional peaks that were not correlated to the canonic oxylipins commonly observed in planktonic diatoms, opening interesting perspectives of possibly finding new oxylipins or other compounds with possible cytotoxic effects.

## Materials and Methods

### Ethics Statement

*Paracentrotus lividus* (Lamarck) were collected from a site in the Bay of Naples that is not privately owned or protected in any way, according to the Italian legislation (DPR 1639/68, 09/19/1980 confirmed on 01/10/2000). Field studies did not include endangered or protected species. All experimental procedures on animals were in compliance with the guidelines of the European Union (Directive 609/86).

### Isolation and morphological identification of two benthic diatoms

Epiphytes were isolated from leaves of *P. oceanica*, collected in Ischia, Naples (Italy) using a sterilized scalpel. Individual diatoms were aspirated by means of a Narishige syringe Syr-12, taking advantage of a Leica micromanipulator under inverted microscopy and transferred into 12-wells multi-wells in sterile seawater. The strains were collected and transferred daily to clean *f/2* medium until monoclonal cultures of the two benthic diatoms were obtained. Diatom cultures were grown in *f/2*medium (Sigma Guillard’s) at 18 °C with a 12:12 photoperiod. Mother cultures of diatoms were transferred every 10 days in new multiwell plates.

Diatom samples from the mother culture were collected, fixed with 2.5% glutaraldehyde, filtered on cellulose Millipore filters and mounted on aluminum stubs for Scanning Electron Microscopy (SEM, Zeiss EVO MA LS). After three washings and treatment with osmium (1%), samples were dehydrated (25, 50, 75 and 100% ethanol) and coated with platinum for SEM observations and morphological identification.

### Molecular characterization of benthic diatoms

Individual cell cultures were collected from the multi-well plates and concentrated by centrifugation for 20 minutes at 4500 rpm (revolution per minute) at 4 °C, then frozen in liquid nitrogen until use. Cell membranes were disrupted by lysis buffer containing Cetyltrimethylammonium bromide (CTAB) 2% and 2-Mercaptoethanol (2-ME, Sigma-Aldrich). RNAse was then added (final concentration 200 μg/ml) and digestion was performed at 65 °C for 45 minutes. Extraction with chloroform/isoamyl alcohol (24:1) was performed two times and 1 volume of ice-cold isopropanol (100%) was then added to the aqueous phase with glycogen for DNA precipitation at −20 °C overnight. After centrifugation, DNA was washed with 75% ethanol, centrifuged for 15 minutes, and air-dried. DNA was suspended in 20 μl sterile water. The amount of total DNA extracted was estimated by measuring the absorbance at 260 nm; purity was calculated using 260/280 and 260/230 nm ratios, using a NanoDrop spectrophotometer (ND-1000 UV-vis Spectrophotometer; NanoDrop Technologies, Wilmington, DE, USA). The integrity of DNA was evaluated by agarose gel electrophoresis.

PCR was performed with specific primers for 18S rRNA (528 F and 1055R^50,51^; see Supplementary Table S1). Additional information is reported in the Supplementary Information file.

### Preparation of cultures

To produce sufficient biomass of the two benthic diatoms, cultures were prepared (using as starting material the monoclonal mother-cultures) in 17-cm glass Petri dishes^22^ and grown for one week. These cultures were then collected by aspiration by means of a sterile Pasteur pipette, and used to prepare massive cultures on a 2% vegetal substrate agar, which is harmless for sea urchins^52^. These massive cultures were grown for one week in a thermostatic chamber, up to the exponential growth phase to facilitate an even diffusion of the diatoms on the whole substrate. When agar massive cultures were inoculated, three glass slides were also deployed on the surface of the agar substratum and, at the end of the exponential phase (1 week), cells grown on the glass slides were counted under an inverted microscope using an ocular micrometer (20×). Based on these counts, the biomass of diatoms fed to sea urchins was then calculated as logC (quantity of intracellular carbon in picograms) = −0.541 + 0.811 × logV (cell volume in μm^3^)^32^.

### Feeding experiments, fertilization and morphological analysis of embryos

Twenty adult (12 females and 8 males) *P. lividus* were reared in each experimental tank of a continuous flow-through system (see Supplementary Information file for further details on the continuous flow-through system) and fed with *Ulva rigida* (3 control replicates) and the two benthic diatoms tested (3 replicates for each species). The daily amount of food (both *Ulva rigida* and the agar substrate incorporating the diatoms) given to sea urchins was 1 gram per sea urchin. After one month of feeding, eggs and sperms were collected from fed sea urchins. Eggs from each female were washed with filtered seawater (FSW) and kept in FSW until use. Concentrated ‘dry’ sperm was collected and kept undiluted at +4 °C until use. Eggs were fertilized utilizing sperm-to-egg ratios of 100:1. Fertilized eggs were kept at 20 °C in a controlled temperature chamber on a 12 h:12 h light:dark cycle. After 48 hours post-fertilization, morphological malformations in sea urchin *plutei* were determined for at least 100 plutei from each female (fixed in formaldehyde 4% in FSW) using a light microscope (ZeissAxiovert 135TV, Carl Zeiss, Jena, Germany). Statistical analyses were performed using GraphPad Prism version 4.00 for Windows (GraphPad Software, San Diego California USA).

Fecal pellets were collected in the tanks during the feeding experiments, concentrated and processed for SEM observations (as described above) in order to detect and confirm the presence of diatoms ingested.

### Embryo collection, RNA extraction, *de novo* transcriptome assembly and Real Time qPCR

After the feeding experiments on adults, about 5000 eggs (in 50 mL of FSW) from each female fed on *U. rigida* and on the two benthic diatoms were collected and fertilized. Embryos were then collected 48 hours post-fertilization (hpf) by centrifugation at 1800 relative centrifugal force (rcf) for 10 minutes in a swing out rotor at 4 °C. Embryos were placed in at least 10 volumes of the RNA*later*^®^, an RNA Stabilization Reagent (Qiagen, Hilden, Germany), and then frozen in liquid nitrogen and kept at −80 °C.

Total RNA was extracted using Aurum^™^ Total RNA Mini Kit (Bio-Rad, Hercules, CA, USA), according to the manufacturer’s instructions^53^ for RNA-seq experiments. Total RNA was extracted using RNAqueous Micro Kit (Ambion from Life Technologies), according to the manufacturer’s instructions for Real Time qPCR experiments^53^. Samples were stored at −80 °C. For each sample, 600 ng of total RNA extracted was retrotranscribed with an iScript™ cDNA Synthesis kit (Bio-Rad, Milan, Italy), following the manufacturer’s instructions.

RNA sequencing was performed (Genomix4Life Srl, Salerno, Italy) on nine samples, grouped in three experimental conditions: Control (*U. rigida*), 1^st^ treatment (*N. shiloi*) and 2^nd^ treatment (*C. closterium*), each one composed of three biological replicates.

Additional Information on preparation of cDNA libraries and raw assembled transcriptome are reported in Supplementary Information file, including Supplementary Figs S1–S4. The full dataset of raw data has been deposited in the SRA database (accession number: SUB2817153).

The expression levels of each gene by Real Time qPCR were analyzed and internally normalized against the control gene for *Pl-Z12-1*^54^ using REST software (Relative Expression Software Tool, Weihenstephan, Germany) based on the Pfaffl method^55,56^. Variation of expression levels were calculated as relative expression ratios of the analyzed genes with respect to control embryos. Only expression levels greater than 1.5-fold with respect to controls were considered significant. Statistical analysis was performed using GraphPad Prism version 4.00 for Windows (GraphPad Software, San Diego, CA, USA). See Supplementary Information for further details.

### ^1^H-NMR Metabolomic analysis of the gonads

For each treatment gonad tissues were collected from five adult sea urchins after one month of feeding and stored at −20 °C. Gonads were re-suspended in 170 µl of H_2_O and 700 µl of methanol and were sonicated for 30 sec. Then, 350 µl of chloroform were added and samples were mixed on an orbital shaker in ice for 10 min. 350 μl of H_2_O/chloroform (1:1, v/v) were added to each cell suspension and centrifuged at 10000 rpm for 10 min at 4 °C. Thereafter, the aqueous (polar) and lipophilic (apolar) phases were collected separately, transferred to a glass vial and dried under nitrogen flow. Samples were analyzed using Nuclear Magnetic Resonance (NMR). The polar fractions were dissolved in 630 µl of PBS-D_2_O with the pH adjusted to 7.20, and 70 µl of sodium salt of 3-(trimethylsilyl)-1-propanesulfonic acid (1% in D_2_O) was used as the internal standard. On the other hand the lipophilic fractions were dissolved in 700 µl of deuterated chloroform. A 600-MHz Bruker Avance DRX spectrometer with a TCI probe was used to acquire ^1^H spectra on the cellular polar fractions. All ^1^H-NMR spectra were acquired at 300 K with the excitation sculpting pulse sequence to suppress water resonance. A double-pulsed field gradient echo was used, with a soft square pulse of 4 ms at the water resonance frequency and with gradient pulses of 1 ms duration, adding 128 transients of 64 k complex points, with an acquisition time of 4 s/transient. Time domain data were all zero-filled to 256 k complex points and an exponential amplification of 0.6 Hz was applied prior to Fourier transformation.

The assignments were based on the comparison of chemical shifts and spin-spin couplings with reference spectra present in the human metabolome database (HMDB)^57^ and the Biological Magnetic Resonance Database (BMRB)^58^.

### Statistical and Pathway Analysis

The 0.50–8.60 ppm spectral region of the ^1^H-NMR spectra was integrated in buckets of 0.04 ppm using the AMIX package (Bruker, Biospin GmbH, Rheinstetten, Germany). The water resonance region (4.5–5.2 ppm) was excluded during the analysis and the bucketed region was normalized to the total spectrum area using Pareto scaling. Orthogonal Projections to Latent Structures discriminant analysis (OPLS-DA) was used to compare the spectra obtained on the polar and apolar phases obtained from gonad tissues after feeding treatments.

## Electronic supplementary material

Supplementary Information
