# Supplementary material for: Toxigenic effects of two benthic diatoms upon grazing activity of the sea urchin: morphological, metabolomic and de novo transcriptomic analysis
Source: Sci Rep. 2018 Apr 4;8:5622. doi: 10.1038/s41598-018-24023-9 (PMC5884808; doi:10.1038/s41598-018-24023-9)

**Supplementary Information**

**Toxigenic effects of two benthic diatoms upon grazing activity of the sea urchin: morphological, metabolomic and *de novo* transcriptomic analysis**

**Nadia Ruocco^1,2,3^, Susan Costantini^4^, Valerio Zupo^5^, Chiara Lauritano^6^, Davide Caramiello^7^, Adrianna Ianora^6^, Alfredo Budillon^4^ Giovanna Romano^6^, Genoveffa Nuzzo^3^, Giuliana D’Ippolito^3^, Angelo Fontana^3^, Maria Costantini^1,^***

*^1^Department of Biology and Evolution of Marine Organisms, Stazione Zoologica Anton Dohrn, Villa Comunale, 80121 Napoli, Italy*

*^2^Department of Biology, University of Naples Federico II, Complesso Universitario di Monte Sant’Angelo, Via Cinthia, 80126, Napoli, Italy*

*^3^Bio-Organic Chemistry Unit, Institute of Biomolecular Chemistry-CNR, Via Campi Flegrei 34, Pozzuoli, Naples 80078, Italy*

*^4^Unità di Farmacologia Sperimentale, Istituto Nazionale Tumori “Fondazione G. Pascale”, IRCCS, Napoli, Italy*

*^5^Center of Villa Dohrn Ischia-Benthic Ecology, Department of Integrative Marine Ecology, Stazione Zoologica Anton Dohrn, P.ta S. Pietro, Ischia, Naples, Italy.*

*^6^Department of Integrative Marine Ecology, Stazione Zoologica Anton Dohrn, Villa Comunale, 80121 Napoli, Italy*

*^7^Unit Marine Resources for Research, Stazione Zoologica Anton Dohrn, Naples, Italy*

**Molecular characterization of benthic diatoms**

The reaction was carried out on a C1000 Touch Thermal Cycler (Applied Biosystem, Monza, Italy) in a final volume of 25 μl containing 300 ng of DNA, 2.5 μl 10x PCR reaction buffer (Roche, Milan, Italy), 2.5 μl 2 mM dNTPs (10x), 20 pmol/µl of each primer, and 0.2 μl 5U/μl Taq (Roche, Milan, Italy). The thermal profile consisted of a denaturation step at 94°C for 3 min, 35 cycles at 94°C for 35 s, 50°C for 35 s and 72°C for 2 min, and a final extension at 72°C for 5min.

The amplified fragment was purified from agarose gel (1.5%) using the QIAquick Gel Extraction kit (Qiagen, Milan, Italy). The purified PCR products were then sequenced on an Applied Biosystems 3730 DNA Analyzer 48 capillaries (Life Technologies) using BigDye^®^ Terminator v3.1 Cycle Sequencing kit (Life Technologies). PCR product sequences obtained with both forward and reverse primers were firstly aligned with the software MultiAlin (http://multalin.toulouse.inra.fr/multalin/) for multiple sequence alignment (Corpet, 1988), in order to reconstruct the total rRNA 18S fragment. The 18S were then aligned using the BLAST algorithm (http://blast.ncbi.nlm.nih.gov/Blast.cgi) to identify the species.

**Experimental rearing for feeding experiments**

A continuous flow-through system was used consisting in nine glass rectangular tanks (35 liters each); seawater was pumped from the sea, collected in an outdoor basin, then filtered twice on gauze filters (200 μ) and moved to an indoor basin. Seawater was filtered again by means of a protein skimmer, a UV sterilizer, a refrigerator and a mechanical filter, and then moved to the experimental tanks aerated by means of air-stones. Water was released through outflow tubes from each tank at a rate of 1 change per hour. The main abiotic parameters were measured three times a week using a multiparametric probe (YSI 85, YSI, Incorporated); seawater was kept at constant temperature (18 ± 1°C), salinity (38 ± 1 %), dissolved O_2_ (7 mg/l) and pH 8.1 using TECO Tr-30 water chillers and Eheim external filters. The tank glasses were cleaned three times a week.

**Samples for *de novo* transcriptome assembly and Real Time qPCR**

For RNA-seq the amount of total RNA extracted was estimated by the absorbance at 260 nm and the purity by 260/280 and 260/230 nm ratios, using a NanoDrop spectrophotometer (ND-1000 UV-Vis Spectrophotometer; NanoDrop Technologies, Wilmington, DE, USA), to exclude the presence of proteins, phenol and other contaminants (Riesgo et al., 2012a). The integrity measurements of RNA were finally assessed by running 100-200 ng of RNA samples in each line of a 6000 Nano LabChip in an Agilent Bioanalyzer 2100 Bioanalyzer (Agilent Technologies, Santa Clara, CA, US). RNA integrity was measured using the RIN value, which is calculated based on the comparison of the areas of 18S rRNA and 28S rRNA (Schroeder et al., 2006). RIN values over a threshold of 8 were considered to indicate non-degraded RNA extraction methods.

For Real Time qPCR synthetized cDNA was used in Real-Time qPCR experiments without dilution in a reaction containing a final concentration of 0.3 mM for each primer and 1× FastStart SYBR Green master mix (total volume of 10 μL) (Applied Biosystems, Monza, Italy). PCR amplifications were performed in a ViiATM7 Real Time PCR System (Applied Biosystems, Monza, Italy) thermal cycler using the following thermal profile: 95 °C for 10 min, one cycle for cDNA denaturation; 95 °C for 15 s and 60 °C for 1 min, 40 cycles for amplification; 72 °C for 5 min, one cycle for final elongation; one cycle for melting curve analysis (from 60 °C to 95 °C) to verify the presence of a single product. Each assay included a no-template control for each primer pair. To capture intra-assay variability, all real-time qPCR reactions were carried out in triplicate. Fluorescence was measured using ViiATM7 software (Applied Biosystems, Monza, Italy).

**De novo transcriptome assembly and data analysis**

RNA samples were used for preparation of cDNA libraries (TruSeq Stranded mRNA) and paired-end sequencing in single (2x100, ~30.000.000 total reads/sample) on Illumina platform Hiseq 2500. Reads were obtained from ends of DNA fragments for ultra-high-throughput sequencing. Prior to further analysis, a quality check was performed on the sequencing data.

A paired-end sequencing was chosen, in which short reads were obtained from ends of DNA fragments for ultra-high-throughput sequencing. Prior to further analysis, a quality check was performed on the sequencing data. The quality of the reads was evaluated also through the FASTQC bioinformatics tool (Andrew 2010; Supplementary Figures S1-S3). The high quality reads from all the samples were joined and then used as input to perform transcriptome assembly, with Trinity (Grabherr et al, 2011).

The full dataset of raw data has been deposited in the SRA database (accession number: SUB2817153).

The raw assembled transcriptome included almost 121 Mbp in 192493 transcripts grouped in 126941 genes. The mean GC content was 41.37%. The average and median contigs lengths were 357 bp and 627 bp, respectively. A transcript is expressed if it has been assembled from RNA-Seq data, but transcription can be quite pervasive, and many transcripts, particularly the very lowly expressed ones have questionable biological significance. Some transcripts may have artificially low (or zero) expression values simply because they are incompletely assembled and not recruited both pairs of Paired End reads were used in order to properly estimate abundance. If we assume that most biologically relevant transcripts are reasonably well assembled and well quantified by the abundance estimation method used, we might infer the approximate number of expressed genes or transcripts as the number that are expressed above some minimum expression threshold.

The sequences of the assembled transcripts were translated into proteins with Transdecoder (http://transdecoder.github.io) (minimum length 100aa). When multiple translations were possible the priority was set in order to obtain the longest complete ORF; when a complete ORF was not detected the longest sequence was kept. A gff3 annotation file was obtained by reporting the coordinates of the CDS for each translated transcript and this was named “Paracentrotus_lividus.gff3”.

To evaluate the correlation between biological replicates and different experimental conditions, the Trinity 'PtR' tool (Grabherr et al, 2011) was adopted. Starting from the counts of each associated feature, this tool performs a counts-per-million (CPM) data transformation followed by a log2 transform. All analyses were performed on *de-novo* genes (Supplementary Figure S4). Reads were mapped to assembled putative transcripts using bowtie (Langmead &Salzberg 2012).

TransDecoder (http://transdecoder.github.io/) software was used to identify candidate coding regions within fragments. ORFs that were at least 100 amino acids long were selected. A total of 72395 ORFs were identified in the analyzed samples.

Expression analysis was performed by RSEM (version 1.1.21) using default parameters, and expression values were converted to FPKM (Fragments per Kilobase of exon per Million fragments mapped; Roberts et al, 2011). Differential expressed analysis was performed by setting a log fold change on base 2 threshold ≥ 1.5 corresponding to a false discovery rate (FRD) < 0.05. Functional annotation analyses were conducted on all differentially expressed genes using Blast2GO.

The Gene Ontology (GO) terms were assigned based on annotation with an E-value of 10^-6^.

**References**

Andrew S. (2010). FastQC, a quality control tool for high throughput sequence data.

Grabherr MG,  Haas BJ, Yassour M, Levin JZ, Thompson DA, Amit I, *et al.* (2011). Full-length transcriptome assembly from RNA-Seq data without a reference genome. *Nat Biotechnol* **29**: 644-652.

Langmead B, Salzberg SL. (2012). Fast gapped-read alignment with Bowtie 2. *Nat Methods* **9:** 357-359.

Roberts A, Trapnell C, Donaghey J, Rinn JL, Pachter L. (2011). Improving RNA-Seq expression estimates by correcting for fragment bias. *Genome Biol* **12**: R22.

**Supplementary Table S1.** Sequences and length of the primers for rRNA 18S, amplicon length and references.

| **Name** | **Sequence (5'→3')** | **Primers Lenght** | **Amplicon Lenght** | **Reference** |
| --- | --- | --- | --- | --- |
| 528F | GCG GTA ATT CCA GCT CCA A | 19 bp | 800 bp | Elwood, 1985  Kooistra et al., 2003 |
| 1055R | ACG GCC ATG CAC CAC CAC CCA T | 22 bp |  |  |

**Supplementary Table S2.** Assignment of the peaks in ^1^H NMR spectra obtained on polar fraction for gonads. In details, the metabolite name and chemical shift has been reported for each peak (expressed in ppm).

| **Metabolites** | **Chemical shift (ppm)** |
| --- | --- |
| isoleucine | 0.92 |
| leucine | 0.94 |
| valine | 0.97 |
| isoleucine | 0.99 |
| valine | 1.02 |
| isoleucine | 1.24 |
| threonine | 1.31 |
| lactate | 1.33 |
| isoleucine | 1.45 |
| alanine | 1.46 |
| arginine | 1.68 |
| leucine | 1.7 |
| lysine | 1.89 |
| isoleucine | 1.96 |
| glutamate | 2.04 |
| glutamate | 2.11 |
| glutamine | 2.13 |
| acetoacetate | 2.28 |
| glutamate | 2.34 |
| glutamine | 2.44 |
| aspartic acid | 2.66 |
| asparagine | 2.84 |
| asparagine | 2.94 |
| lysine | 3.02 |
| histidine | 3.16 |
| Choline | 3.19 |
| arginine | 3.23 |
| tryptophan | 3.29 |
| Proline | 3.33 |
| glucose | 3.39 |
| Proline | 3.41 |
| glucose | 3.46 |
| Choline | 3.51 |
| glucose | 3.52 |
| Glycine | 3.54 |
| threonine | 3.57 |
| valine | 3.60 |
| glucose | 3.73 |
| alanine | 3.76 |
| glucose | 3.82-3.88 |
| asparagine | 4 |
| Choline | 4.06 |
| lactate | 4.11 |
| ATP | 4.211 |
| threonine | 4.244 |
| ATP | 4.28 |
| ATP | 4.50 |
| ATP | 4.60 |
| glucose | 5.22 |
| ATP | 6.13 |
| tyrosine | 6.88 |
| histidine | 7.09 |
| tyrosine | 7.17 |
| tryptophan | 7.19-7.31 |
| Phenylalanine | 7.32-7.42 |
| tryptophan | 7.72 |
| histidine | 7.9 |
| ATP | 8.53 |

**Supplementary Table S3.** Assignment of the peaks in ^1^H NMR spectra obtained in the lipophilic fraction of gonads. In details, the metabolite name, group and chemical shift has been reported for each peak (expressed in ppm).

| **Metabolites** | **Group** | **Chemical shift** |
| --- | --- | --- |
| Cholesterol | C_18_**H**_3_ | 0,67 |
| Cholesterol | C_26_**H**_3_,C_27_**H**_3_ | 0,88 |
| Fatty acid residues | ω-C**H**_3_ | 0,89 |
| Fatty acid residues | ω-C**H**_3_ in omega-3 | 0,98 |
| Cholesterol | C_19_**H**_3_ | 1,01 |
| Cholesterol | Esterified C_19_**H**_3_ | 1,03 |
| Cholesterol | Multiple cholesterol protons | 1,06-1,09 |
| Fatty acid residues | (C**H**_2_)_n_ | 1,3 |
| Fatty acid residues | COCH_2_-C**H**_2_ | 1,6 |
| Fatty acid residues | β-C**H**_2_ of arachidonic and eicosapentaenoic acids | 1,68 |
| Fatty acid residues | -C**H**_2_-CH= | 2,04 |
| Fatty acid residues | -CO-C**H**_2_ | 2,3 |
| Fatty acid residues | α and β C**H**_2_ of docosahexaenoic acid | 2,37-2,39 |
| Fatty acid residues | -CH=CH-C**H**_2_-CH=CH- of linoleic acid | 2,76 |
| Fatty acid residues | (CH=CH-C**H**_2_-CH=CH)_n<1_ | 2,8 |
| Phospholipids | (-CH_2_-NH_2_) of phoshatidylethanolamine | 3,11-3,14 |
| Phospholipids | (-C**H**_2_-N-(CH_3_)_3_) of sphingomyelin and phospahtidylcholine | 3,33 |
| Choline | N(C**H**_3_)_3_ | 3,38 |
| Cholesterol | C_3_**H** | 3,5 |
| Triglycerides | C_1_**H** and C_3_**H** of glycerol | 4,15 |
| Triglycerides | C_1_**H** and C_3_**H** of glycerol | 4,29 |
| Phosphatidylcholine | POCH2 | 4,33-4,43 |
| Glycerophospholipid | CHOCOR | 5,15-5,22 |
| Triglycerides | C_2_**H** of glycerol | 5,25 |
| Fatty acid residues | C**H**=C**H** | 5,36 |
| Cholesterol | C_6_**H** | 5,37 |

**Supplementary Table S4.** Data of expression levels in embryos deriving from sea urchins fed with *C. closterium* and *N. shiloi* were reported as a fold difference (in red up-expressed genes; in light blue down-expressed genes) from control (represented by embryos deriving from adults of sea urchins fed with *U. rigida*) at 48 hpf . Fold differences greater than ± 1.5 were considered significant. The genes were divided in the four functional classes (stress, skeletogenesis, development and differentiation and detoxification according the **Supplementary Figure S7**).

|  |  | ***C. closterium*** |  | ***N. shiloi*** |
| --- | --- | --- | --- | --- |
| **Stress** | *hsp70* | 1.71 |  | 2.25 |
|  | *hsp60* | 2.23 |  | 2.36 |
|  | *hsp56* | 2.59 |  | -3.40 |
|  | *MTase* | -1.66 |  | -3.14 |
|  | *GS* | 2.77 |  | 1.87 |
|  | *cytb* | 3.37 |  | 2.76 |
|  | *p38 MAPK* | 0.33 |  | -0.75 |
|  | *14-3-3* ε | 3.99 |  | 2.93 |
|  | *caspase 3/7* | -1.18 |  | -2.45 |
|  | *CASP8* | 2.74 |  | 1.36 |
|  | *NF-kB* | -1.80 |  | 3.00 |
|  | *p53* | -3.74 |  | -3.48 |
|  | *HIF1A* | -1.98 |  | -2.75 |
|  | *ERCC3* | 0.95 |  | -0.31 |
|  |  |  |  |  |
| **Skeletogenesis** | *SM30* | -2.11 |  | -3.79 |
|  | *SM50* | 1.91 |  | 0.79 |
|  | *BMP5-7* | -1.74 |  | -3.86 |
|  | *Nec* | 2.76 |  | 3.14 |
|  | *uni* | -3.44 |  | -2.72 |
|  | *p16* | 1.17 |  | 1.21 |
|  | *p19* | 3.60 |  | 4.83 |
|  | *C-jun* | 2.83 |  | 2.47 |
|  |  |  |  |  |
| **Development/Differentiation** | *hat* | -1.32 |  | 1.08 |
|  | *sox9* | 2.04 |  | 0.98 |
|  | *BP10* | -1.28 |  | -1.25 |
|  | *Blimp* | 2.28 |  | 1.63 |
|  | *Alix* | 0.27 |  | 0.74 |
|  | *Wnt5* | 1.06 |  | 0.91 |
|  | *Wnt6* | 1.94 |  | 1.56 |
|  | *Wnt8* | 1.83 |  | -1.24 |
|  | δ*-2-catenin* | -2.05 |  | -2.91 |
|  | *nodal* | 2.88 |  | 2.84 |
|  | *tcf4* | -0.81 |  | 0.46 |
|  | *TCF7* | 2.31 |  | -2.88 |
|  | *FoxG* | 1.98 |  | 2.22 |
|  | *FOXA* | -3.29 |  | -1.75 |
|  | *Foxo* | 2.12 |  | 2.55 |
|  | *GFI1* | -3.11 |  | -2.96 |
|  | *OneCut* | 4.83 |  | 4.87 |
|  | *TAK1* | 4.27 |  | -2.38 |
|  | *VEGF* | -3.00 |  | -2.85 |
|  | *JNK* | 0.87 |  | 0.74 |
|  |  |  |  |  |
| **Detoxification** | *MT* | 4.63 |  | 3.45 |
|  | *MT4* | 1.13 |  | 4.13 |
|  | *MT5* | 2.06 |  | 4.49 |
|  | *MT6* | -2.36 |  | 3.67 |
|  | *MT7* | 2.79 |  | 3.59 |
|  | *MT8* | -2.59 |  | -2.90 |
|  | *MDR1* | 2.75 |  | 2.98 |
|  | *CAT* | 1.78 |  | 1.64 |

**Supplementary Table S5.** Common upragulated and downregulated genes in Venn diagrams (see **Figure 8**), comparing the groups “control (*U. rigida*, feeding control) versus *N. shiloi*”, “control versus *C. closterium*” and “*N. shiloi* versus *C. closterium*”.

| **Compared groups** | **Upregulated genes** | **Downregulated genes** |
| --- | --- | --- |
|  |  |  |
| **“control vs *N. shiloi*”** | PREDICTED: uncharacterized protein LOC580508 | 40S ribosomal S9 |
| **and** | dynein alpha flagellar outer arm | PREDICTED: uncharacterized protein LOC582117 |
| **“control vs C. closterium** | PREDICTED: uncharacterized protein LOC582863 | glutathione S-transferase omega-like 2 |
|  | CMRF35-like molecule 1 | PREDICTED: uncharacterized protein LOC105439325 |
|  | pyruvate dehydrogenase E1 component subunit mitochondrial | cytosolic beta-glucosidase |
|  | laminin subunit alpha |  |
|  | intraflagellar transport 56 |  |
|  | eukaryotic translation initiation factor 3 subunit L |  |
|  | PREDICTED: uncharacterized protein LOC100889257 |  |
|  | hydroxysteroid dehydrogenase 2 |  |
|  | CDP-diacylglycerol--inositol 3-phosphatidyltransferase |  |
|  | craniofacial development 2-like |  |
|  | RNA-directed DNA polymerase from mobile element jockey-like |  |
|  | cleavage stimulation factor subunit 1 |  |
|  | zinc finger CCCH domain-containing 13 |  |
|  | nmrA-like family domain-containing 1 |  |
|  | von Willebrand factor type EGF and pentraxin domain-containing 1-like |  |
|  | PREDICTED: uncharacterized protein LOC580397 |  |
|  | PREDICTED: enkurin |  |
|  |  |  |
|  |  |  |
| **“control vs *N. shiloi*”** | N-terminal EF-hand calcium-binding 1 | RNA-directed DNA polymerase from mobile element jockey-like |
| **“control vs *C. closterium*”** | T-complex 1 subunit epsilon | Retrovirus-related Pol poly from transposon partial |
| **and “*N. shiloi* vs *C. closterium*”** | PREDICTED: uncharacterized protein K02A2.6-like |  |
|  | 39S ribosomal mitochondrial |  |
|  |  |  |
|  |  |  |
| **“control vs *N. shiloi*”** | 40S ribosomal partial | ATP synthase subunit mitochondrial |
| **and “*N. shiloi* vs *C. closterium*”** | beta-lactamase domain-containing 2 | seleno W2 |
|  | metallo ase inhibitor 2 | guanosine-3 ,5 -bis(diphosphate) 3 -pyrophosphohydrolase MESH1 |
|  | arrestin domain-containing 1 |  |
|  | peptidyl-tRNA hydrolase mitochondrial |  |
|  |  |  |
| **“control vs *C. closterium*”** | [Nematostella vectensis] | T-complex 1 subunit epsilon |
| **and “*N. shiloi* vs *C. closterium*”** | annexin A13 | PREDICTED: uncharacterized protein LOC590579 |
|  | transglutaminase-like protein | mucin partial |
|  | ribonucleotide reductase small partial | nmrA-like family domain-containing 1 |
|  | proteasome subunit beta type-4 | 39S ribosomal mitochondrial |
|  | PREDICTED: myosin-IIIb | general transcription factor IIH subunit 1 |
|  | brachyury | signal peptidase complex subunit 3 |
|  | maternal Vg1 | [Nematostella vectensis] |
|  | DNA replication factor Cdt1 | PREDICTED: uncharacterized protein LOC575620 isoform X2 |
|  | cytosolic beta-glucosidase | high-affinity choline transporter 1-like |
|  | armadillo repeat-containing 3 |  |
|  | ATP synthase subunit mitochondrial |  |
|  | 60S acidic ribosomal P1 |  |
|  | PREDICTED: uncharacterized protein LOC100892278 isoform X1 |  |
|  | apoptotic chromatin condensation inducer in the nucleus isoform X2 |  |
|  | PREDICTED: ropporin-1-like protein |  |
|  | succinyl- ligase [GDP-forming] subunit mitochondrial |  |
|  | regulator of chromosome condensation |  |
|  | tolloid 1 |  |
|  | carboxypeptidase B |  |
|  | laminin epi-1 |  |
|  | Retrovirus-related Pol poly from transposon opus |  |
|  | cytochrome c oxidase subunit 6C [Macaca fascicularis] |  |
|  | PREDICTED: uncharacterized protein LOC576204 isoform X1 |  |
|  | transmembrane 19 |  |
|  | PREDICTED: uncharacterized protein LOC100369247 |  |
|  | isocitrate dehydrogenase [NADP] mitochondrial-like |  |
|  | cytosolic non-specific dipeptidase |  |
|  | gem-associated 2 |  |
|  | PREDICTED: LOW QUALITY PROTEIN: uncharacterized protein LOC586069 |  |
|  | GMP reductase 1 isoform X2 |  |
|  | inorganic pyrophosphatase |  |
|  | aminoacylase-1 isoform X2 |  |
|  | fibropellin partial |  |
|  | PREDICTED: alpha-L-fucosidase |  |
|  | PREDICTED: uncharacterized protein C7orf26 homolog |  |
|  | PREDICTED: uncharacterized protein LOC756005 |  |
|  | RNA polymerase II subunit |  |
|  | dynactin subunit 6 |  |
|  | PREDICTED: mimitin, mitochondrial |  |
|  | WD repeat-containing 66 |  |
|  | plexin domain-containing 2 |  |
|  | E3 ubiquitin- ligase CHIP |  |
|  | ubiquitin carboxyl-terminal hydrolase BAP1 isoform X3 |  |
|  | PREDICTED: uncharacterized protein LOC590790, partial |  |
|  | arf-GAP with Rho-GAP ANK repeat and PH domain-containing 1 isoform X10 [Strongylocentrotus purpuratus] | |
|  | PREDICTED: uncharacterized protein LOC100892549 |  |
|  | tectonin beta-propeller repeat-containing 2 isoform X2 |  |
|  | PREDICTED: uncharacterized protein LOC105443887 |  |
|  | heterogeneous nuclear ribonucleo s A1 homolog |  |
|  | nucleolin isoform X3 |  |
|  | DNA-directed RNA polymerase II subunit RPB7 |  |
|  | UDP-N-acetylglucosamine--peptide N-acetylglucosaminyltransferase 110 kDa subunit isoform X1 | |
|  | 3-oxoacyl-acyl-carrier- reductase-like |  |
|  | NADH dehydrogenase [ubiquinone] 1 alpha subcomplex subunit 6 |  |
|  | PREDICTED: uncharacterized protein LOC757164 isoform X3 |  |
|  | heat shock partial |  |
|  | bifunctional D-cysteine desulfhydrase 1-aminocyclopropane-1-carboxylate mitochondrial [Strongylocentrotus purpuratus] | |
|  | actin-related 6 |  |
|  | unconventional myosin-IXa |  |
|  | pre-mRNA-splicing factor ATP-dependent RNA helicase PRP1 |  |
|  | very long-chain specific acyl- mitochondrial |  |
|  | nascent polypeptide-associated complex subunit muscle-specific form isoform X2 | |
|  | proteasome maturation |  |
|  | cystathionine gamma-lyase |  |

**Supplementary Figure S1.** Overview of the sequence quality for ‘control’ (feeding *U. rigida*) samples. On the left the green dots mean that the sample' reads pass that specific analytical step, while the red dots mean that the sample' reads failed. The orange dots mean that analytical step is ok, but with lower quality. On the right is represented a box plot for each cycle.


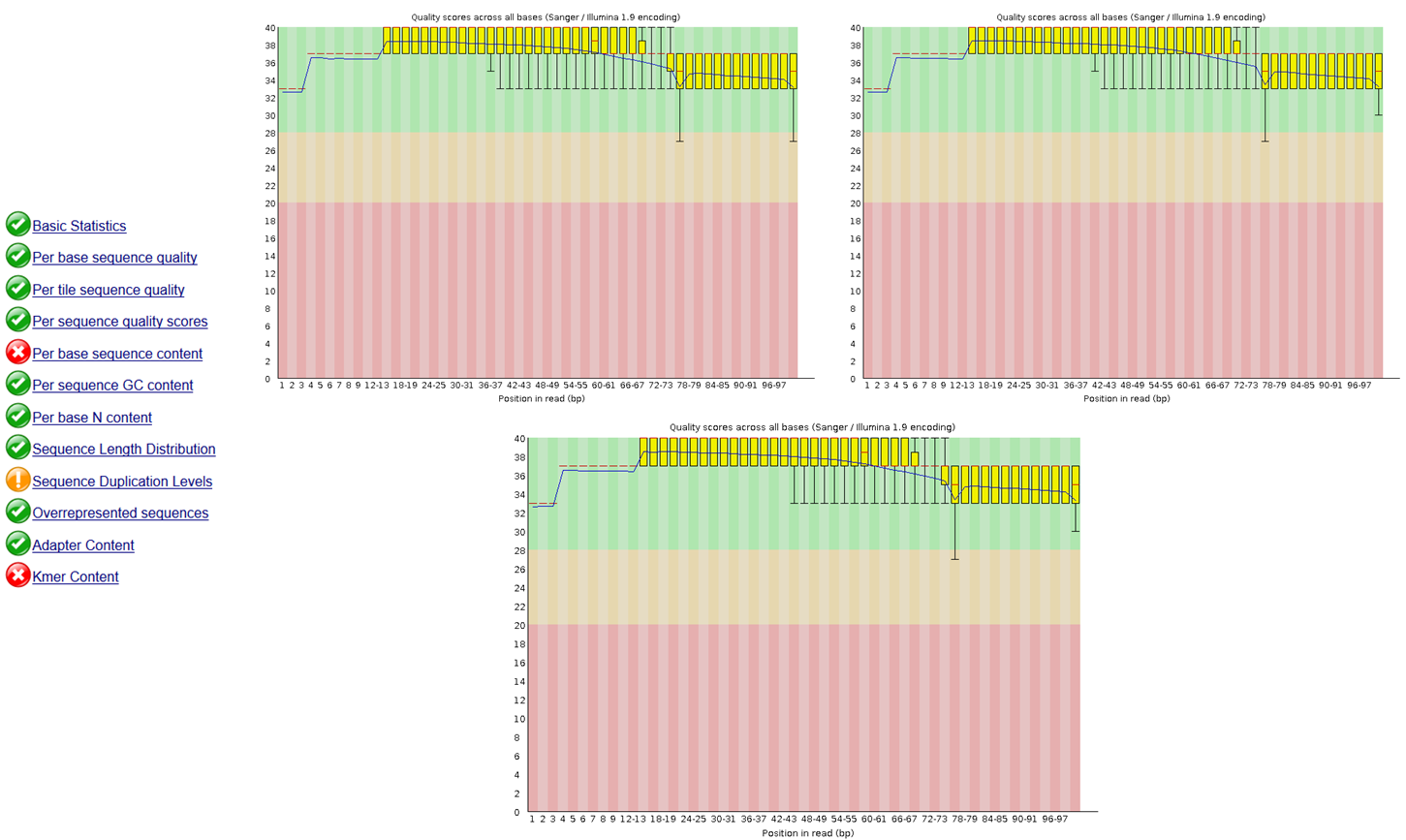


**Supplementary Figure S2.** Overview of the sequence quality for ‘1st treatment’ (feeding with *N. shiloi*) samples. For further details see legend to the **Supplementary Figure S1**.


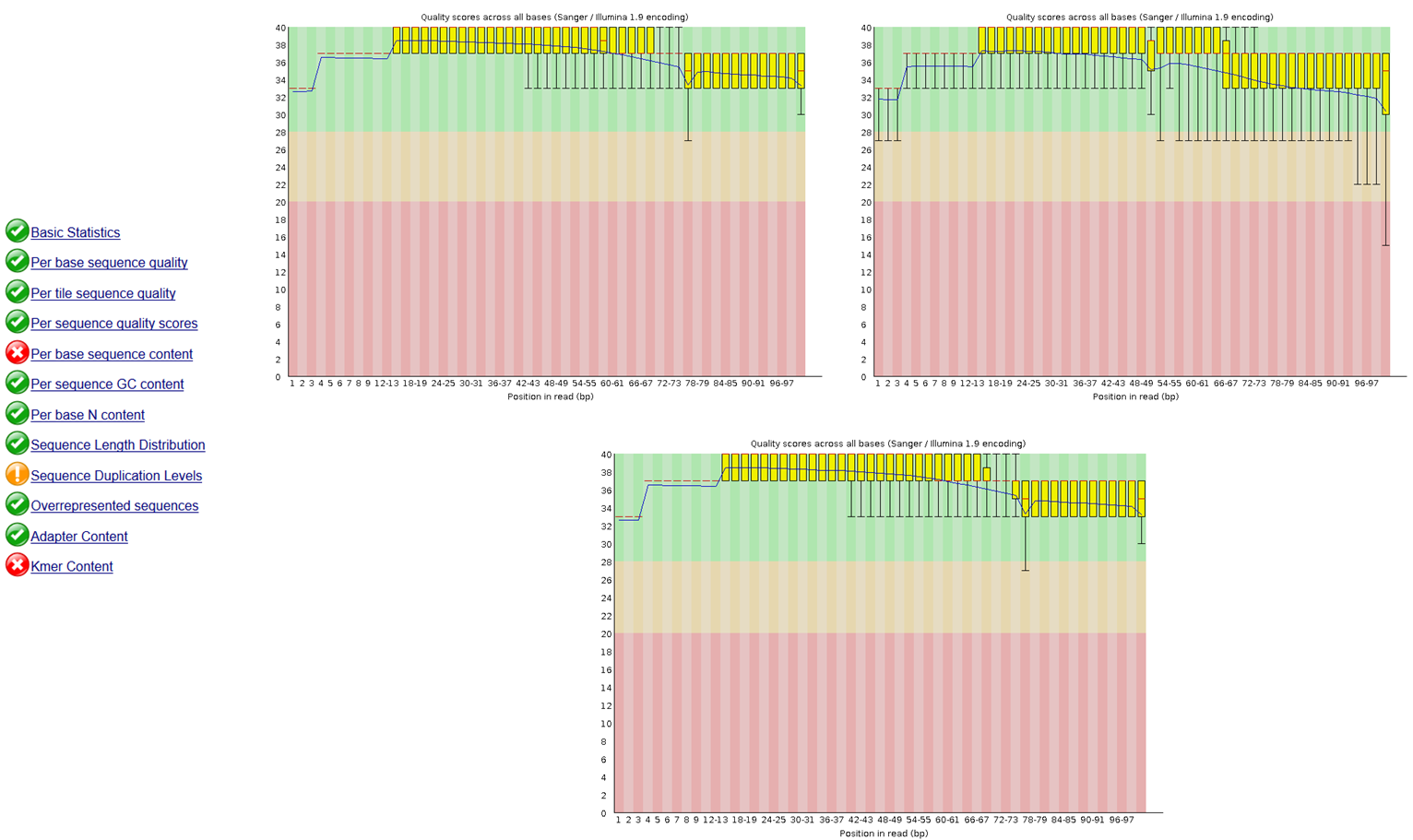


**Supplementary Figure S3.** Overview of the sequence quality for ‘2nd treatment’ (feeding with *C. closterium*) samples. For further details see legend to the **Supplementary Figure S1**.


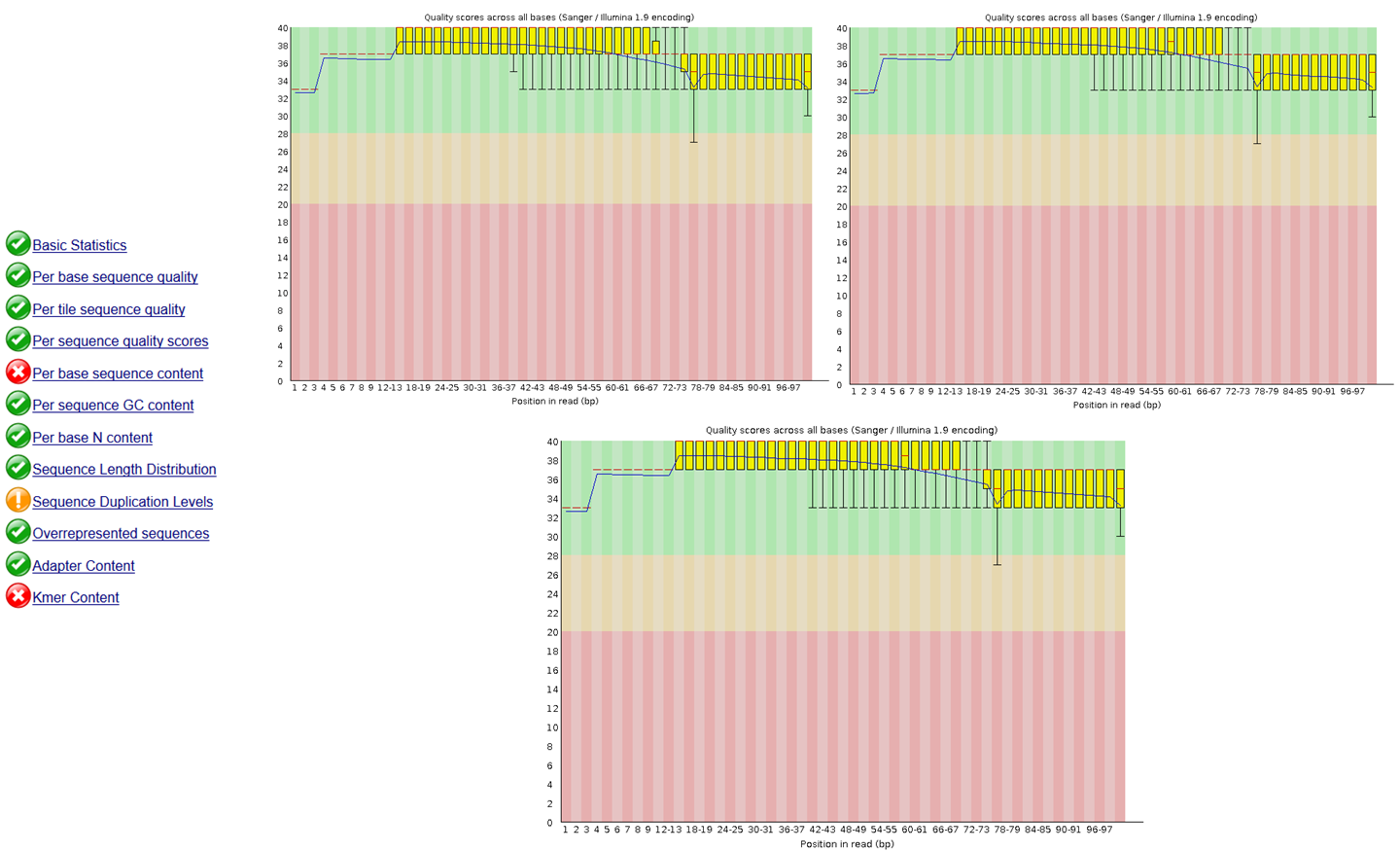


**Supplementary Figure S4.** Sum of fragments for all analyzed samples (grouped by experimental conditions: ‘control’ (feeding *U. rigida*), ‘1st treatment’ (feeding with *N. shiloi*) and ‘2nd treatment’ (feeding with *C. closterium*).

**
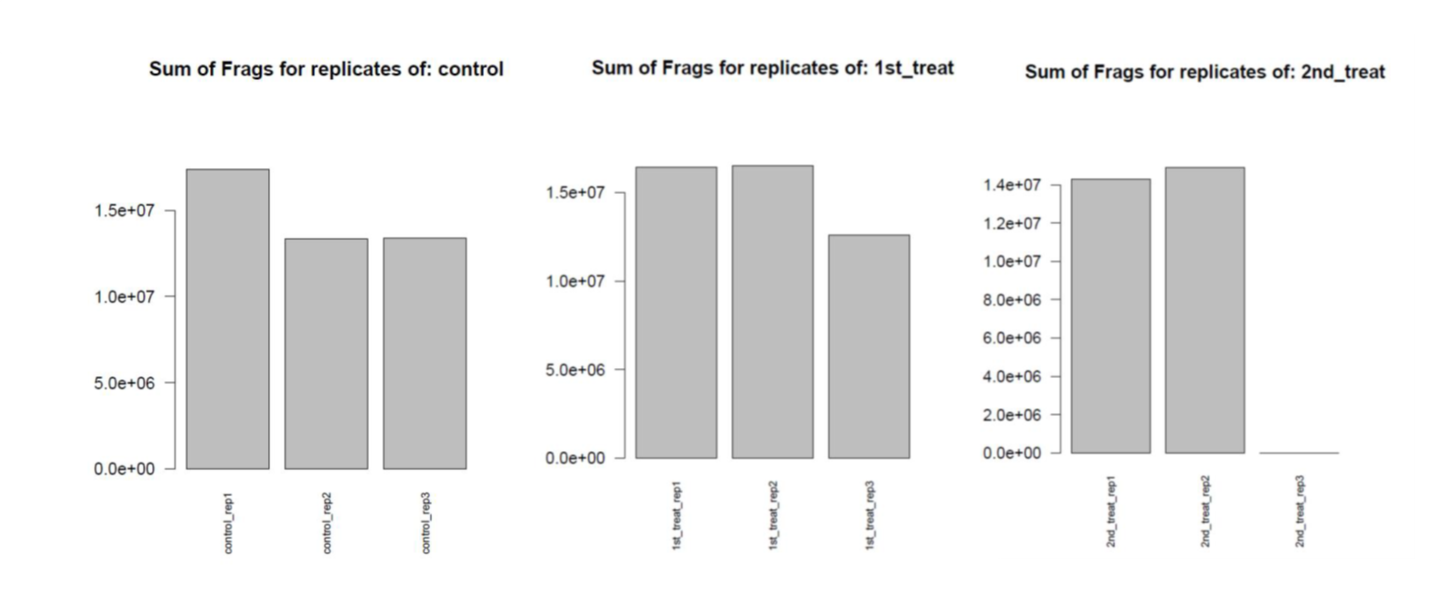
**

**Supplementary Figure S5.** Examples of malformations observed in *P. lividus* plutei spawned from adults fed for one month with *N. shiloi* and *C. closterium* (B-I) in comparison with (A) control embryos spawned from adults fed with *U. rigida*. Such plutei showed (B) a poorly-formed apex with (C-D) spicules that appeared crossed at the apex or disjoined at the tip, or with (3D) poorly-formed and degraded arms. Some embryos showed (E-G) an abnormal trunk, and (H) delayed (gastrula stage) and (I) abnormal development.


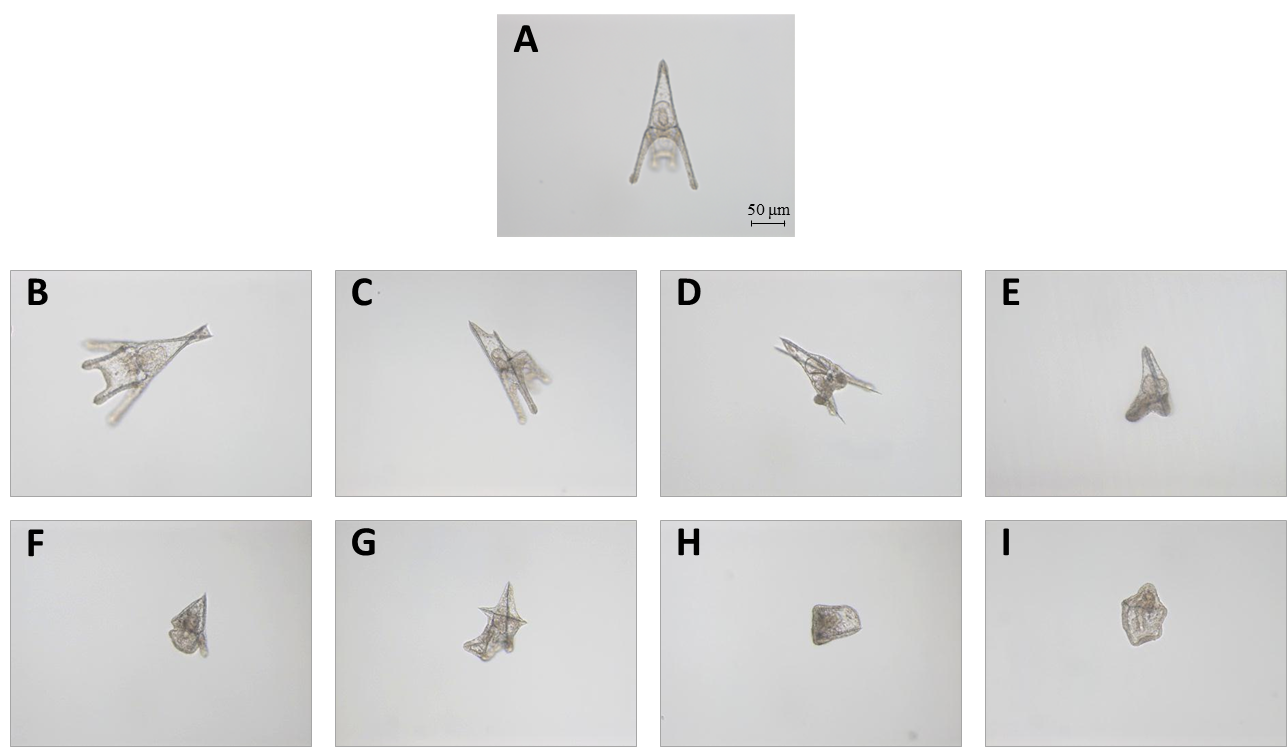


**Supplementary Figure S6.** Scanning electron micrographs (SEM) of (A-E) fecal pellets from adult sea urchins fed with the benthic diatom *C. closterium*. Scale bar = 1 μm.


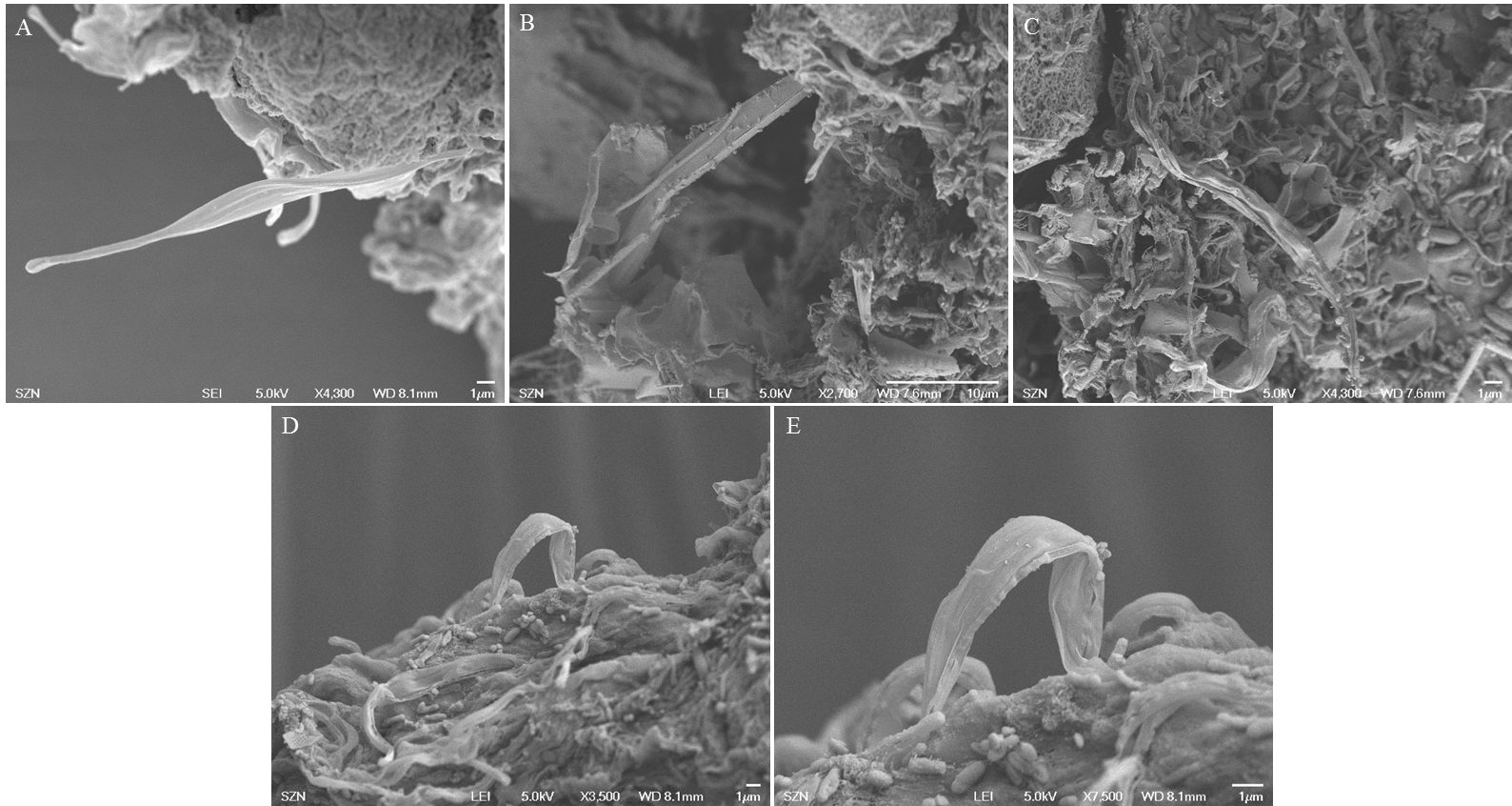


**Supplementary Figure S7.** The scheme indicate the four functional classes of genes used to which the fifty genes analyzed in the present study belong: canonical stress genes, skeletogenic genes, genes involved in developmental and differentiation processes and genes involved in detoxification processes.

**
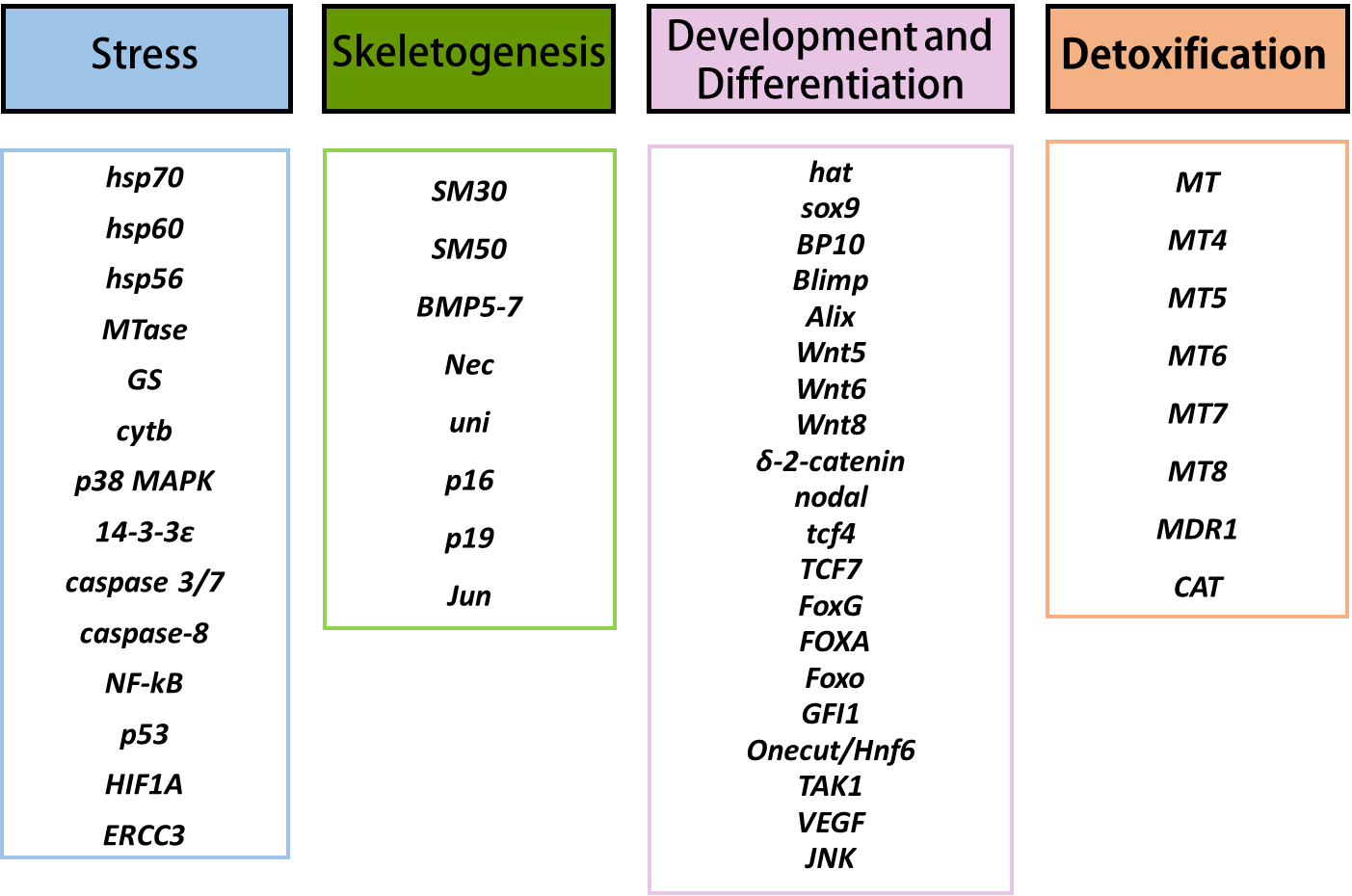
**

**Supplementary Figure S8**. Real-Time qPCR at pluteus stage. Histograms show the differences in expression levels of analyzed genes involved in different embryonic processes (see also Supplementary Figure S6) followed by Real Time qPCR. *P. lividus* adults were fed with *U. rigida*, *N. shiloi* *and C. closterium*. After one month of feeding, gametes were collected, fertilized and embryos were grown until the pluteus stage (48 hpf). Data are reported as a fold difference compared with control (mean ± SD) embryos in sea water without PUAs. Fold differences greater than ± 1.5 were considered significant (see also **Figure 7** and **Supplementary Table S4** for the values).


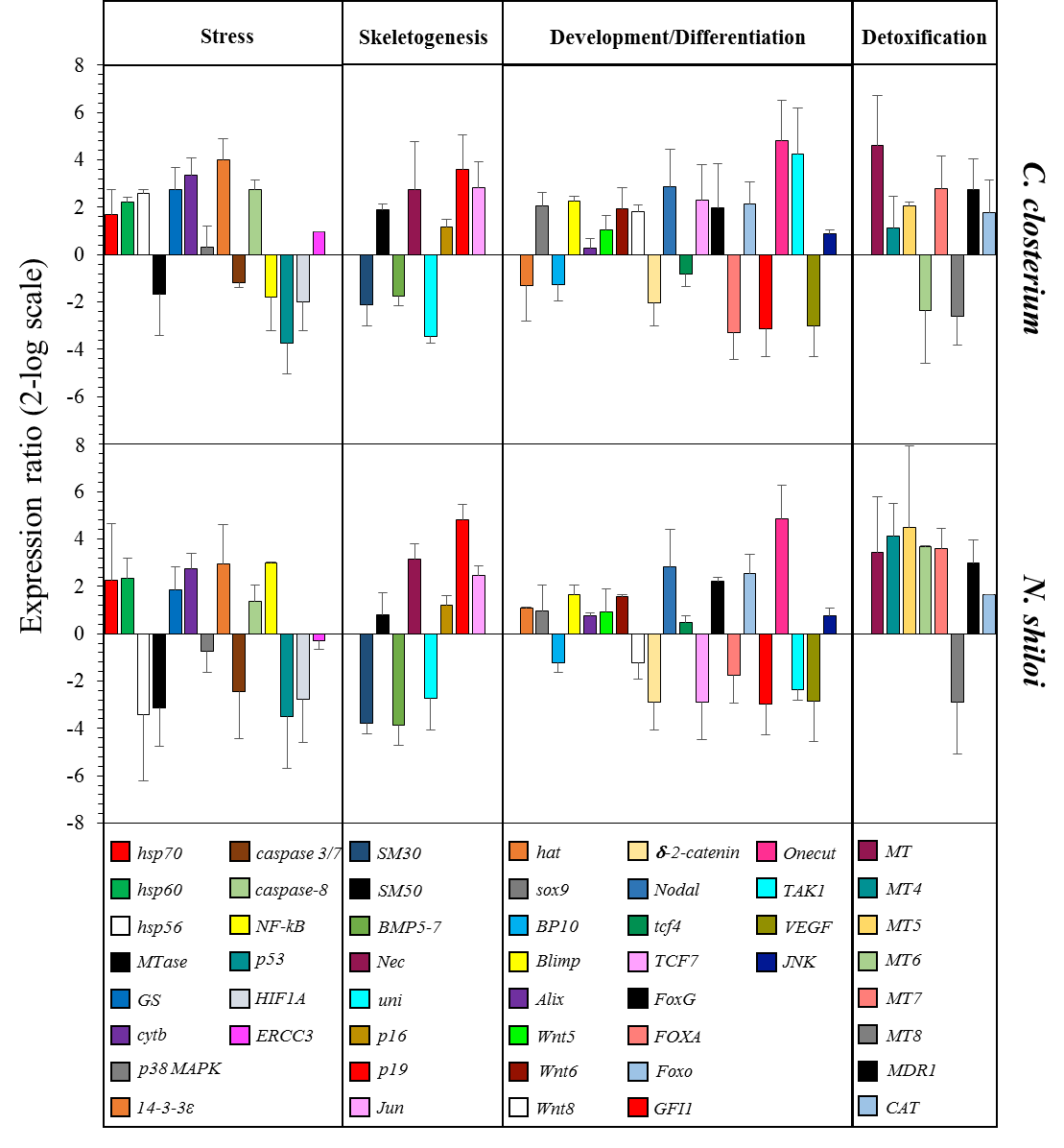


**Supplementary Figure S9.** Examples of five genes (*FoxG*, *catenin*, *cytochrome*, *metallotionein* and *14-3-3ε*) common to Real Time qPCR and RNA-seq experiments. Fold differences greater than ± 1.5 were considered significant.


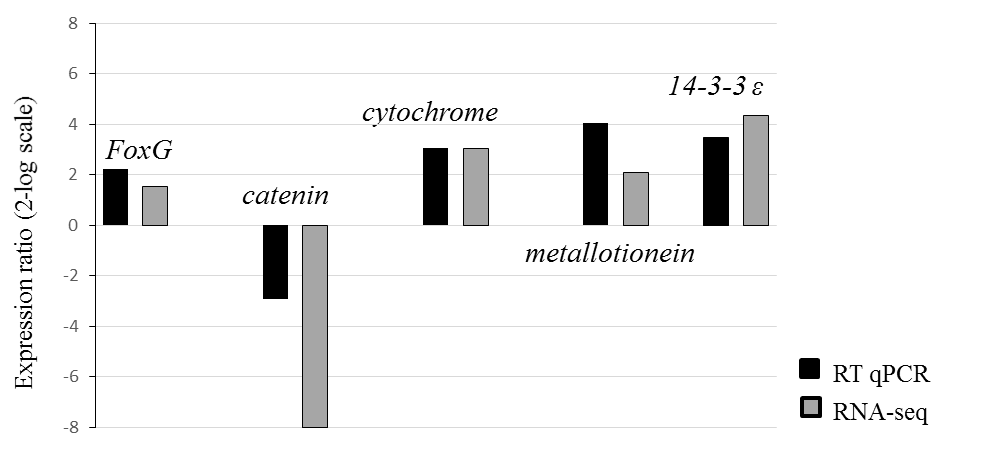

Supplement: Supplementary file 1 — Supplementary Information [file 41598_2018_24023_MOESM1_ESM.docx]
